# Spatial patterns of congruence or mismatch between taxonomic, functional, and phylogenetic diversity and endemism of perennial flora along the aridity gradient of Chile

**DOI:** 10.3389/fpls.2024.1418673

**Published:** 2024-08-30

**Authors:** Paola Poch, Elie Poulin, María Fernanda Pérez, Gioconda Peralta, Luis Felipe Hinojosa

**Affiliations:** ^1^ Institute of Ecology and Biodiversity (IEB), Santiago, Chile; ^2^ Departamento de Ciencias Ecológicas, Facultad de Ciencias, Universidad de Chile, Las Palmeras, Santiago, Chile; ^3^ Departamento de Ecología, Facultad de Ciencias Biológicas, Pontificia Universidad Católica de Chile, Santiago, Chile

**Keywords:** environmental filter, ecological opportunity, environmental heterogeneity, geographic isolation, eco-evolutionary mechanisms

## Abstract

**Introduction:**

Understanding the relationships between taxonomic, functional, and phylogenetic diversity and endemism across environmental gradients is essential for elucidating the eco-evolutionary mechanisms that shape local plant communities.

**Methods:**

A database was compiled from field surveys, national herbarium records, and virtual records of perennial plant specimens collected in the aridity gradient of northern Chile, between 18 and 32°S. A large-scale dated phylogeny of available perennial plants was used, and 11 functional traits were selected to construct a dendrogram using the Unweighted Pair-Group Method with Arithmetic Mean (UPGMA) method for the species present in our database. We calculated spatial patterns of a-diversity, including taxonomic (TD), functional (FD), and phylogenetic (PD) diversity, as well as weighted (WE), functional (FE), and phylogenetic (PE) endemism. We used multiscale geographically weighted regression (MGWR) to identify spatial congruencies and discrepancies among these dimensions and to test different eco-evolutionary processes.

**Results:**

The diversity indices TD, FD and PD showed similar geographic patterns (R_2_ > 0.93), with lower diversity observed in absolute desert regions. The pattern of weighted endemism (WE) showed a weak association with functional endemism (FE) and phylogenetic endemism (PE) (local R_2_ < 0.48). The regions with lower FD or PD than expected given the TD (i.e. FD<TD and PD<TD) are mainly located in desert areas, as well as in high Andean areas influenced by the Atacama Desert, suggesting communities with associated *in situ* speciation processes, as well as a limitation of morpho-functional trait diversity in response to extreme environmental conditions (environmental filter hypothesis). Similarly, where FE and PE values are higher than expected given the WE (i.e. FE>WE and PE>WE), they are found in arid, high Andean and transitional zones, at different altitudes, which would indicate a greater presence of phylogenetic lineages and species with morpho-functional traits related to extreme environmental conditions and transitional biomes (arid-semiarid).

**Discussion:**

These spatial discrepancies suggest different eco-evolutionary drivers between the dimensions of diversity and endemism (taxonomic, functional, and phylogenetic). Areas of high diversity and high endemism do not necessarily coincide, and both should be addressed by conservation efforts.

## 1 Introduction

Understanding the processes and mechanisms that shape the distribution of biodiversity along environmental gradients remains a central question in ecology and biogeography (Hawkins, 2001; Kreft and Jetz, 2007; Xu et al., 2019). Traditional metrics for measuring diversity (e.g., taxonomic diversity (TD) and phylogenetic diversity (PD)) or endemism (e.g., weighted endemism (WE) and phylogenetic endemism (PE)) (Schall and Pianka, 1978; Faith, 1992; Rosauer et al., 2009) treat all species as morpho-functionally equivalent (Tilman, 2001; Petchey and Gaston, 2006; Mammola et al., 2021). To address these limitations, diversity metrics that incorporate functional trait information have been developed to provide initial insight into the role of ecological processes in community assembly (Petchey and Gaston, 2006; Skeels and Yaxley, 2023).

The functional dimension provides information on the different roles or functions of species in ecosystems as inferred from morpho-physiological traits (Swenson, 2011; Volaire et al., 2020). Functional diversity (FD) integrates the variability of traits in a set of species, with high diversity of functional traits providing stability through the coexistence of different strategies, allowing communities to respond effectively to changing environments (Petchey and Gaston, 2006; Cadotte et al., 2011; Ochoa-Ochoa et al., 2020). Conversely, areas of endemism are essential because they capture facets of biodiversity that are not represented elsewhere. Functional endemism (FE), analogous to PE, describes areas with high proportions of ecologically distinct but narrowly distributed species (Skeels and Yaxley, 2023).

Recent studies have assessed multiple diversity dimensions (TD, PD, and FD) and endemism (WE and PE) in plants, showing linear covariation at global and regional scales (Scherson et al., 2017; Le Bagousse-Pinguet et al., 2019; Zhou et al., 2019; Doxa et al., 2020). However, these metrics may exhibit low spatial association or mismatch at local scales, reflecting different evolutionary (e.g., speciation, extinction, dispersal) and ecological (e.g., environmental filtering, competition) mechanisms shaping spatial variation in plant diversity and endemism (Doxa et al., 2020; Qian et al., 2021; Freitas et al., 2021). Various eco-evolutionary processes based on ecological opportunity, habitat filtering, environmental heterogeneity, biotic exchange, and geographic isolation have been proposed to explain mechanisms in areas where these measures are incongruent (Simpson, 1945; Zobel, 1997; Chesson, 2000; Voskamp et al., 2017; Mráz et al., 2016).

The ecological opportunity hypothesis proposes that rapid diversification occurs in novel environments with ample space and low competitive pressure, resulting in high TD and FD and low PD (Simpson, 1945; Wellborn and Langerhans, 2015). Conversely, the environmental filter hypothesis proposes low FD in sites with limited ecological space or restrictive conditions (Keddy, 1992; Grime, 2006). The environmental heterogeneity hypothesis suggests greater phenotypic differentiation with greater spatial variation in water and solar energy availability (Newbery et al., 1996).

Higher PD, indicating communities of distantly related species compared to TD, may result from increased immigration due to biotic exchange or ancient lineages with few distinct species (Voskamp et al., 2017; Isaac et al., 2007; Davies and Buckley, 2011). On the other hand, several studies report a consistent correlation between WE and PE, possibly due to local speciation or climatic refugia harboring endemic species (Mráz et al., 2016; Keppel et al., 2012; Mishler et al., 2014). However, studies of plant communities with restricted distributions of functional traits, or functional endemism (FE), and their spatial correlation with WE and PE remain scarce (Skeels and Yaxley, 2023).

### 1.1 Arid and semi-arid zones of northern Chile

**Figure 1:**
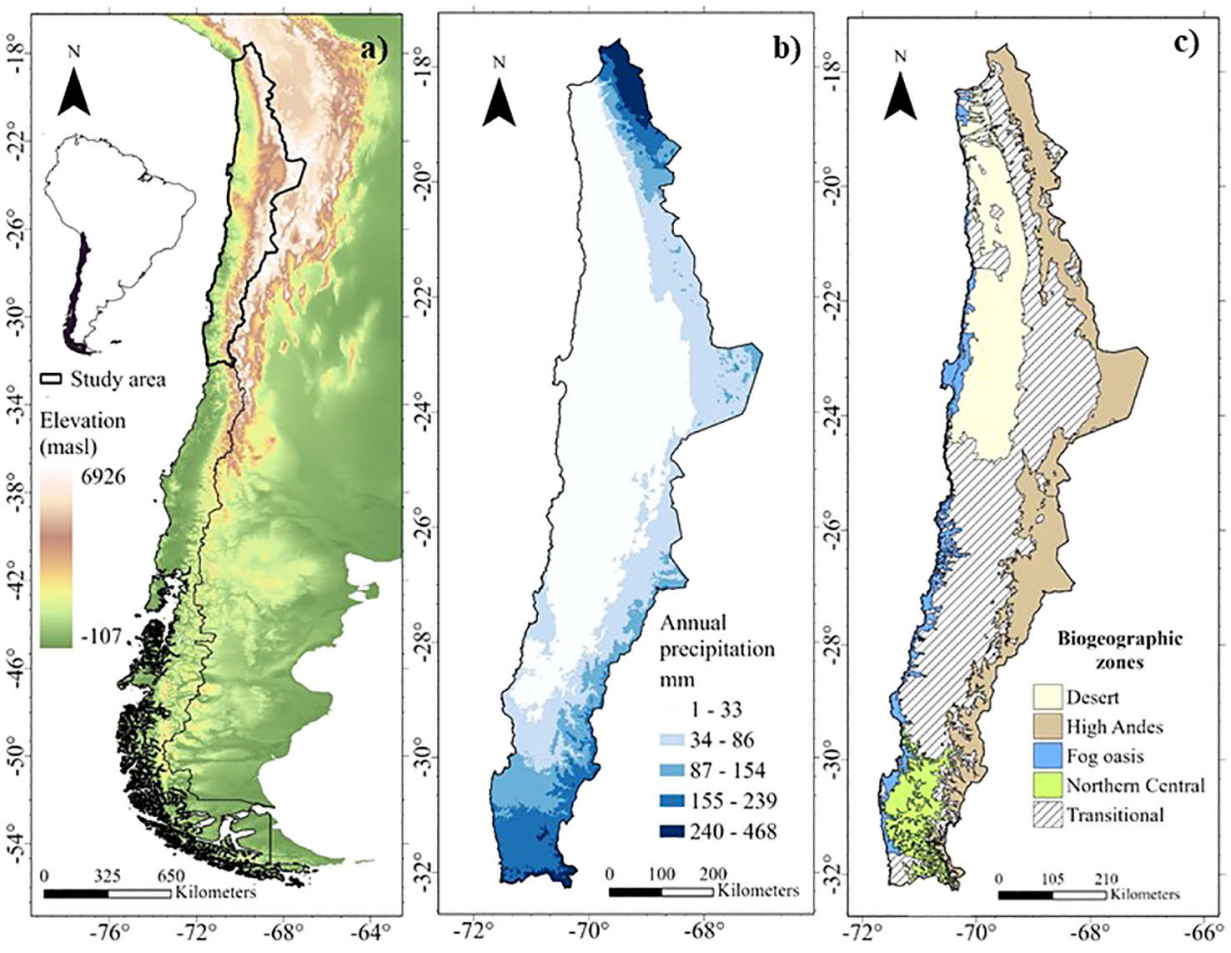
Environmental gradients of altitude, precipitation, and biogeographic areas in the arid and semi-arid zones of northern Chile. **(A)** Altitude gradient of Chile (Farr et al., 2007); **(B)** Precipitation gradient in the arid and semi-arid zones of northern Chile (Fick and Hijmans, 2017) y **(C)** Biogeographic areas in the arid and semi-arid zones of northern Chile.

The arid and semi-arid zones of northern Chile (18 to 33°S) offer unique opportunities to evaluate biogeographic hypotheses related to latitudinal and altitudinal gradients in the distribution of diversity and endemism patterns of perennial plant species. This region, located in an altitude range from the coastal plains to over 3,000 masl in the Andes, has a pronounced climatic variation along altitudinal and latitudinal gradients, between arid and semi-arid climates, with precipitation of great spatial and temporal variability (annual averages below 300 mm) (Garreaud et al., 2003; Sarricolea and Romero, 2015). Four main ecosystems can be identified (**Figure 1**):

i) The Atacama Desert: Known as the driest subtropical desert in the Southern Hemisphere. Lower TD and PD (related lineages) have been observed in this area as a result of *in situ* speciation processes associated with aridity (Scherson et al., 2017). In addition, we expect lower observed FD in response to restrictive environmental conditions that limit functional trait space (*environmental filters hypothesis*).
ii) Fog oasis: The coastal fog, known as “camanchaca”, supports a unique and diverse flora in ecosystems discontinuously distributed along the coastal mountain range, like islands in the arid region, characterized by high endemism (Rundel et al., 1991; Cavieres et al., 2002; Dillon, 2005). We expect to find high values and correlations between WE, PE and FE (*geographic isolation hypothesis*).
iii) Northern High Andes of Chile or Puna: Northern distribution zone, between 18 and 32°S, above the tree line along the Chilean Andes (based on Testolin et al., 2020), characterized by a relatively recent (5-10 Ma) geological origin (Hughes and Eastwood, 2006; Luebert and Weigend, 2014; Pérez-Escobar et al., 2022) and is located on a steep climatic gradient, with annual precipitation of about 160 mm in the north (18°S), decreasing to 30 mm towards 23°S due to the influence of the Atacama Desert (Villagrán et al., 1983; Arroyo et al., 1988; Rundel et al., 2002; Karger et al., 2017). The current floristic composition is characterized by its diversity (Arroyo et al., 1998; Rundel et al., 2002; Luebert and Gajardo, 2000). We expect to observe high TD and FD and low PD due to diversification in novel environments with ample space and low competitive pressure, especially in the north (18°S) with higher rainfall (*ecological opportunity hypothesis*).
iv) Northern Central Chile: The climate of central Chile, between 30 and 32°S, varies from arid to semi-arid, with a north-south moisture gradient, including winter precipitation conditions (<300 mm) (Garreaud et al., 2003). It is a transition zone between the Mediterranean and arid zones (Miller, 1976), an ecotone between the last sclerophyllous forest of central Chile and the dry scrub and succulent formations of the southern Atacama Desert, resulting in high diversity (Bull-Hereñu et al., 2005; Guerrero et al., 2011). We expect to observe higher PD compared to TD, indicating distantly related species communities, which may be the result of increased immigration due to biotic exchange or old lineages with few distinct species (*biotic exchange hypothesis).* In addition, we expect higher FD compared to the other diversity dimensions due to greater spatial variation in the availability of water and solar energy (*environmental heterogeneity hypothesis*).

The purpose of this paper is to provide answers to the following questions: How are multiple dimensions of diversity and endemism correlated spatially (latitude and altitude)? Where do discrepancies between these measures occur? Understanding the mechanisms underlying the diversity of the perennial flora of arid and semi-arid northern Chile will be advanced by examining the spatial correlations and discrepancies in diversity and endemism measures. Summary predictions based on the hypotheses and mechanisms behind these patterns are described in **Table 1**.

**Table 1:** Predictions of spatial incongruence or congruence between diversity and endemism measures.

Hypothesis	Mechanism	Predictions Area	Relationship between metrics
Ecological opportunity	High *in situ* speciation	Desert, Puna and high Andean	PD < TD
Ecological opportunity	High phenotypic differentiation driven by wide ecological space	Puna and high Andean	PD < FD
Environmental filter	Restrictive environmental conditions that would limit functional trait space	Desert	FD < TD
Environmental filter	Restrictive environmental conditions that would limit functional trait space	Desert	FD < PD
Environmental heterogeneity	Niche partitioning	Northern Central Chile (27-33°S)	FD > TD
Biotic interchange	High immigration and low *in situ* speciation rate	Northern Central Chile (27-33°S)	PD > TD
Geographic isolation	Low dispersal or geographic isolation	Fog Oases and high Andean	Correlation between WE, PE and FE

## 2 Materials and methods

### 2.1 Study area and species occurrence data

The study area comprises the arid and semi-arid zones of Chile, located between 18 and 32°S and covering approximately 302,039 km^2^, and includes the main ecosystems in latitude and altitude (Atacama Desert, Fog Oasis, Puna and North Central Chile) (**Figure 1**). The perennial flora was used as a model because of its temporal persistence and different drought tolerance mechanisms. Perennial species with arboreal, shrub, sub-shrub, and succulent growth forms were considered according to the classification of the Catalogue of the vascular plants of Chile by Rodriguez et al. (2018), non-native species were excluded (**Supplementary Table S1**). The selection of growth forms follows Taylor et al. (2023), which establishes the importance of shrubs and sub-shrubs in the world’s main arid and Mediterranean biomes. In addition, succulents are included due to their high endemism and importance in Chile’s arid and semi-arid zones (Guerrero et al., 2011).

A dataset of occurrence data was compiled from field surveys, national herbarium records (AGUCH, CONC, MNHN, and ULS), and virtual databases (e.g., Global Biodiversity Information Facility-GBIF). Occurrences were corrected by eliminating outlier records based on known ranges from the literature. We compiled a total of 117,926 unique occurrence records for 716 native perennial species of Chile (out of a total of 851 species) (**Supplementary Table S1**), with 115,475 records from the herbarium, 1,700 records from field surveys, and 751 records from GIF, which were reprojected into a WGS84 coordinate system (EPSG:4326). We used ‘dplyr’ (Wickham et al., 2023) for database query and management, ‘sf’ (Pebesma and Bivand, 2023) and ‘terra’ (Hijmans, 2024) for spatial data analysis.

### 2.2 Biodiversity analyses and associated statistical tests

#### 2.2.1 Grid cell size selection

The choice of grid cell size was based on redundancy values. Redundancy is calculated as 1- [richness/(number of samples)] (Garcillán et al., 2003). Redundancy ranges from 0 to 1. A value close to one represents good overall species sampling, while a value of zero means that there is only one sample per species, thus poor sampling. An average redundancy (ARd) of 0.6 is considered acceptable (Thornhill et al., 2016; 2017; Luebert et al., 2022). We conducted the analyses at four spatial resolutions: 25, 50, 75 and 100 km grid cells. We found that a 25 km x 25 km grid cell was the optimal size for our dataset (**Supplementary Figures S1**, **S2**). The cells with low redundancy (less than 0.6) were interpolated using Bayesian Kriging with a search neighborhood of 1 to 2 cells in ArcgisPro 3.1.2 (ESRI, 2023).

#### 2.2.2 Alpha diversity metrics

Taxonomic diversity (TD) was calculated as the number of species in each grid cell (Laffan et al., 2008). Weighted Endemism (WE) was calculated as the sum of the number of species present in each cell in a local neighborhood, weighted by the fraction of the area they inhabit (Laffan and Crisp, 2003), with high values indicating centers of endemism.

A global dated phylogenetic reconstruction of vascular plants available in Smith and Brown (2018) was used to derive the evolutionary criteria. Species present in the study area but absent in the phylogenetic reconstruction were added to their respective genera using the approach of Qian and Jin (2016) and Jin and Qian (2023) have been commonly used in studies on phylogenetic diversity and structure in regional and global floras (e.g. Yue and Li, 2021; Zhang et al., 2021; Cai et al., 2023; Qian et al., 2024). 206 genera (out of a total of 236 genera present in this study) are represented in the Smith and Brown (2018) phylogeny (**Supplementary Table S2**). We pruned all species with no records information (**Supplementary Figure S3**). For this we used ‘U.PhyloMaker’ package (Jin and Qian, 2023). Phylogenetic diversity (PD) was estimated as the sum of branch lengths found on each cell, considering a branch as the minimum distance connecting each species to the most recent common ancestor. Higher PD values indicate greater local phylogenetic divergence (Faith, 1992). Phylogenetic endemism (PE) describes the extent to which unique phylogenetic lineages are limited to restricted geographic areas, and is defined as the total branch length of the phylogenetic tree of lineages present in a grid cell divided by the ranges of those lineages (Rosauer et al., 2009), with high values indicating centers of endemic evolutionary origins.

We used the functional diversity (FD) approach proposed by Petchey and Gaston (2006), which is based on a dendrogram representing the functional relationships shared by species. A matrix of traits per species was constructed to construct the dendrogram from which FD is measured. We selected 11 traits to represent different axes of functional strategy of the flora with respect to the climatic gradient of temperature and precipitation (**Supplementary Table S3**). The traits were weighted according to their functional importance for drought resistance, from 0 to 1, with a higher weight indicating better plant tolerance to drought. The functional trait weighting proposed by Larraín-Barrios et al. (2018) for coastal desert plants in the Atacama region of Chile was taken into account (**Supplementary Tables S3, S4**). We calculated the Gower (1966) similarity coefficient, which was converted to a dissimilarity distance matrix (D = 1-S) and subjected to hierarchical cluster analysis using the UPGMA method to generate a dendrogram (Petchey and Gaston, 2006) (**Supplementary Figure S4**). This dendrogram represents the similarity of traits among species and is used as a functional analog of phylogeny in PD. For this we used cluster package (Maechler et al., 2021).

The FD index was estimated by trait dendrogram analysis, defined as the sum of branch lengths of a functional dendrogram constructed by cluster analysis (Petchey and Gaston, 2006). FD was calculated for each grid cell. This metric does not require abundance data, only records of species occurrence, and is well suited for predicting ecosystem functioning (Petchey and Gaston, 2006; Zeng et al., 2019). Functional endemism (FE) reflects how restricted to a geographic range species with particular functional traits are found (Skeels and Yaxley, 2023), which is an adaptation of the PE proposed by Rosauer et al. (2009). This adaptation involves replacing branch lengths from the phylogeny with branch lengths from the functional dendrogram (UPGMA). High values would indicate species centers with particular functional trait sets in a specific region.

The 716 native perennial species were considered for all analyses (**Supplementary Table S1**). All metrics of diversity and endemism were calculated using the Dinamica EGO 7.8 software and the Biodinamica 2.2 package (Oliveira et al., 2019) in the R programming language.

#### 2.2.3 Association and geographic mismatches between different dimensions of diversity and endemism

The relationships between the different dimensions of diversity and endemism of the perennial flora of the arid and semi-arid zone of Chile were evaluated using Multiscale Geographically Weighted Regression (MGWR) (Fotheringham et al., 2017). Six models were run to explore the relationships between the dimensions of diversity as well as endemism, and the percentage of variation explained by each size was evaluated: 1) FD ~ TD, 2) PD ~ TD, 3) FD ~ PD, 4) WE ~ FE, 5) WE ~ PE, and 6) FE ~ PE. Local regressions (R^2^) were calculated for each model, assuming a Gaussian function with a bandwidth obtained by a “golden section search” based on minimizing the value of the Akaike information criterion (AICc). The distribution of local fits (local R^2^) generated by the MGWR provides insight into the spatial variation of the relationships between the diversity and endemism dimensions. An area with a higher local R^2^ indicates a more significant association between pairs of variables (i.e., TD ~ FD, TD ~ PD, and FD ~ PD). In addition, MGWR residuals were plotted to assess the magnitude and direction of the discrepancy or mismatch between the different measures of diversity and endemism along the latitude and altitude of the study area, to test the hypotheses described above and in **Table 1**. For example, FD ~ PD is a positive residual indicating sites where ecological opportunity and competitive interactions facilitate trait diversification (e.g., FD > PD), and negative residuals indicate where FD is limited by habitat filtering (e.g., FD < PD). ArcgisPro 3.1.2 (ESRI, 2023) software was used.

## 3 Results

### 3.1 Floristic composition in the arid and semi-arid northern Chile

The perennial flora of the arid and semi-arid north of Chile is composed of 43 orders, 75 families, 236 genera and 851 species (**Supplementary Table S1**). The floristic composition is unbalanced in the tree of life, as ten plant families represent more than 73% of all species evaluated, while the remaining 65 families represent less than 26.4% of plant diversity. In particular, the Asteraceae families represent about 30.6%, being predominant in the arid and semi-arid ecosystems of Chile (**Table 2**). The 10 genera with the highest species diversity represent 40.2% of the floristic composition. The genus *Senecio* was the most representative with 11.6% (99 species). On the other hand, the most common life form is shrub with 52.0% (443 species) and sub-shrub with 28.5% (243 species) (**Supplementary Table S1** and **Supplementary Figure S5**).

**Table 2:** Number of species recorded for the ten most common families and genera present in the perennial flora of arid and semiarid northern Chile.

Family	Species number	Percent (%)	Genus	Species number	Percent (%)
Asteraceae	260	30.6	*Senecio*	99	11.6
Cactaceae	114	13.4	*Adesmia*	46	5.4
Fabaceae	89	10.5	*Eriosyce*	39	4.6
Solanaceae	34	4	*Haplopappus*	35	4.1
Verbenaceae	30	3.5	*Copiapoa*	34	4
Boraginaceae	26	3.1	*Nolana*	22	2.6
Nolanaceae	22	2.6	*Heliotropium*	19	2.2
Calceolariaceae	18	2.1	*Calceolaria*	18	2.1
Chenopodiaceae	18	2.1	*Baccharis*	16	1.9
Apiaceae	15	1.8	*Atriplex*	14	1.6
**Total**	**626**	**73.6**	**Total**	**342**	**40.2**

### 3.2 Diversity and endemism patterns

Of the total number of species inventoried (851 species), functional and phylogenetic information was obtained for 716 native species, representing 84.1% of the total flora recorded. For all subsequent analyses, these 716 native perennial species were considered (**Supplementary Table S1**).

**Figure 2:**
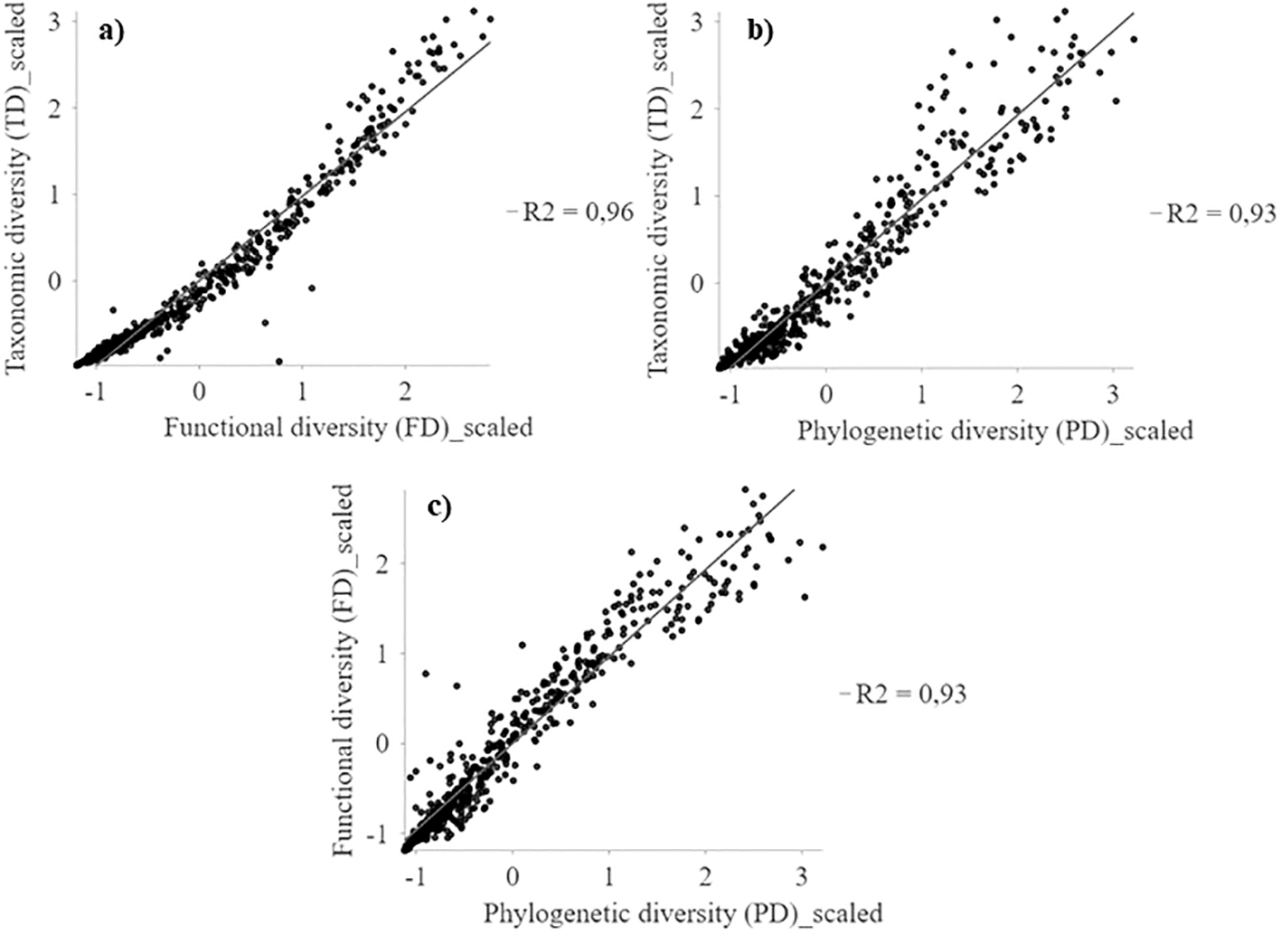
Multiscale geographically weighted regression (MGWR) between TD, FD and PD spatial patterns of the perennial flora of northern Chile. **(A)** TD~FD; **(B)** TD~PD, and **(C)** FD~PD.

**Figure 3:**
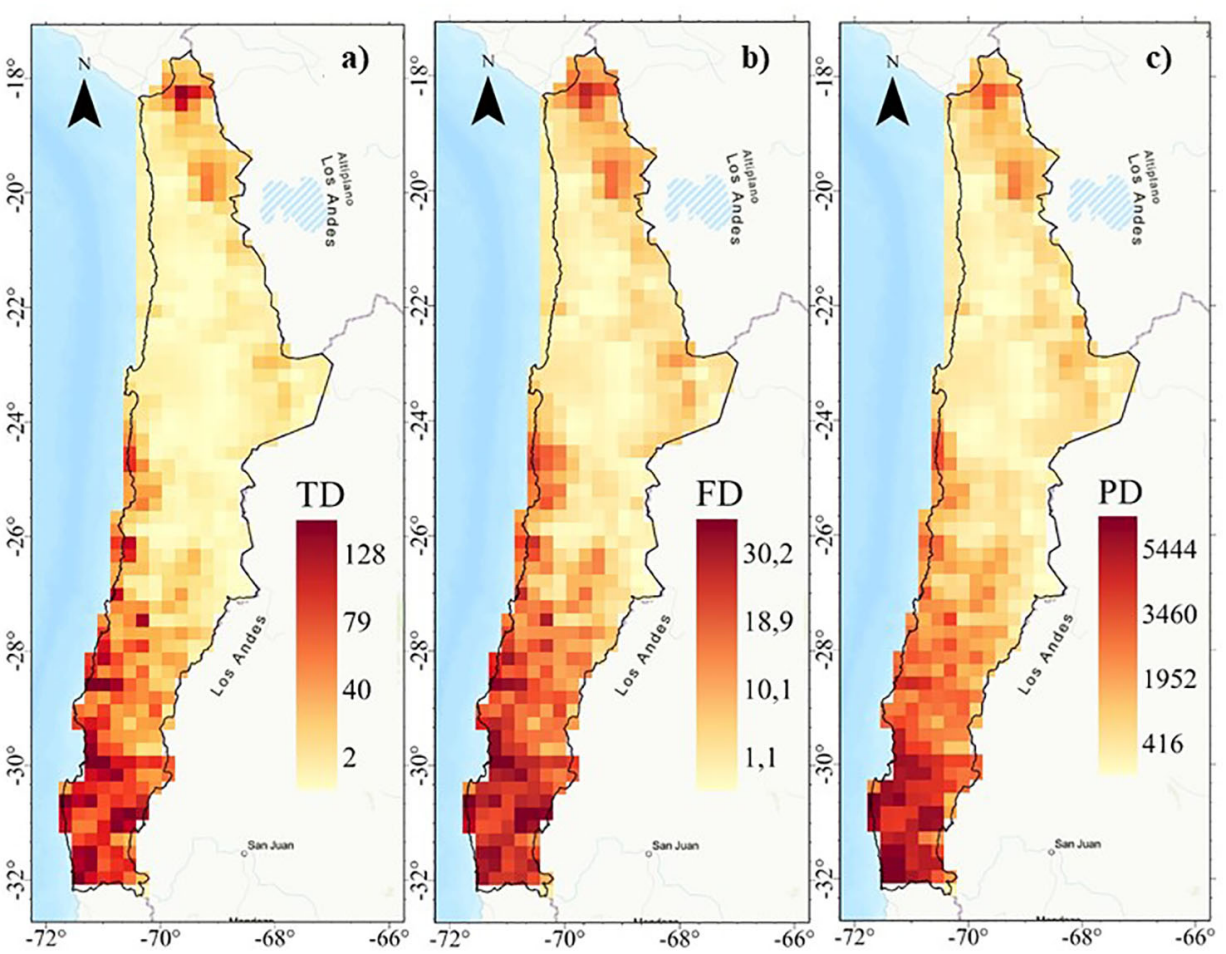
Spatial patterns of diversit y: **(A)** Taxonomic diversity (TD), **(B)** Functional diversity (FD), and **(C)** Phylogenetic diversity (PD) of the perennial flora of northern Chile.

Taxonomic (TD), functional (FD) and phylogenetic (PD) diversity indices show similar geographic patterns (r^2^ > 0.93; **Figure 2**) with a positive latitudinal gradient from north to south for all diversities (**Supplementary Figure S6**). The highest diversity values are concentrated in the highlands of northern Chile (18°S) and coastal areas between 24° and 32°S, extending southward from the coast to the mountains from 28°S (**Figure 3**, **Supplementary Figure S6**). Regions of high diversity (TD, FD, and PD) correspond to areas with higher rainfall or available moisture for plants, such as the mountainous areas of northern Chile (18°S), semi-arid zones, pluvial-seasonal transition zones between 27° and 33°S, and coastal fog oasis areas south of 24°S. Conversely, desert areas show low diversity (**Figure 3**). The highest TD in a single cell was 128, while the FD reached a maximum of 30,2 and the PD was 5,444. In addition, regardless of the spatial resolution (e.g., 100 and 75 km grid), the spatial patterns of TD, FD, and PD are consistent (**Supplementary Figure S7**).

**Figure 4:**
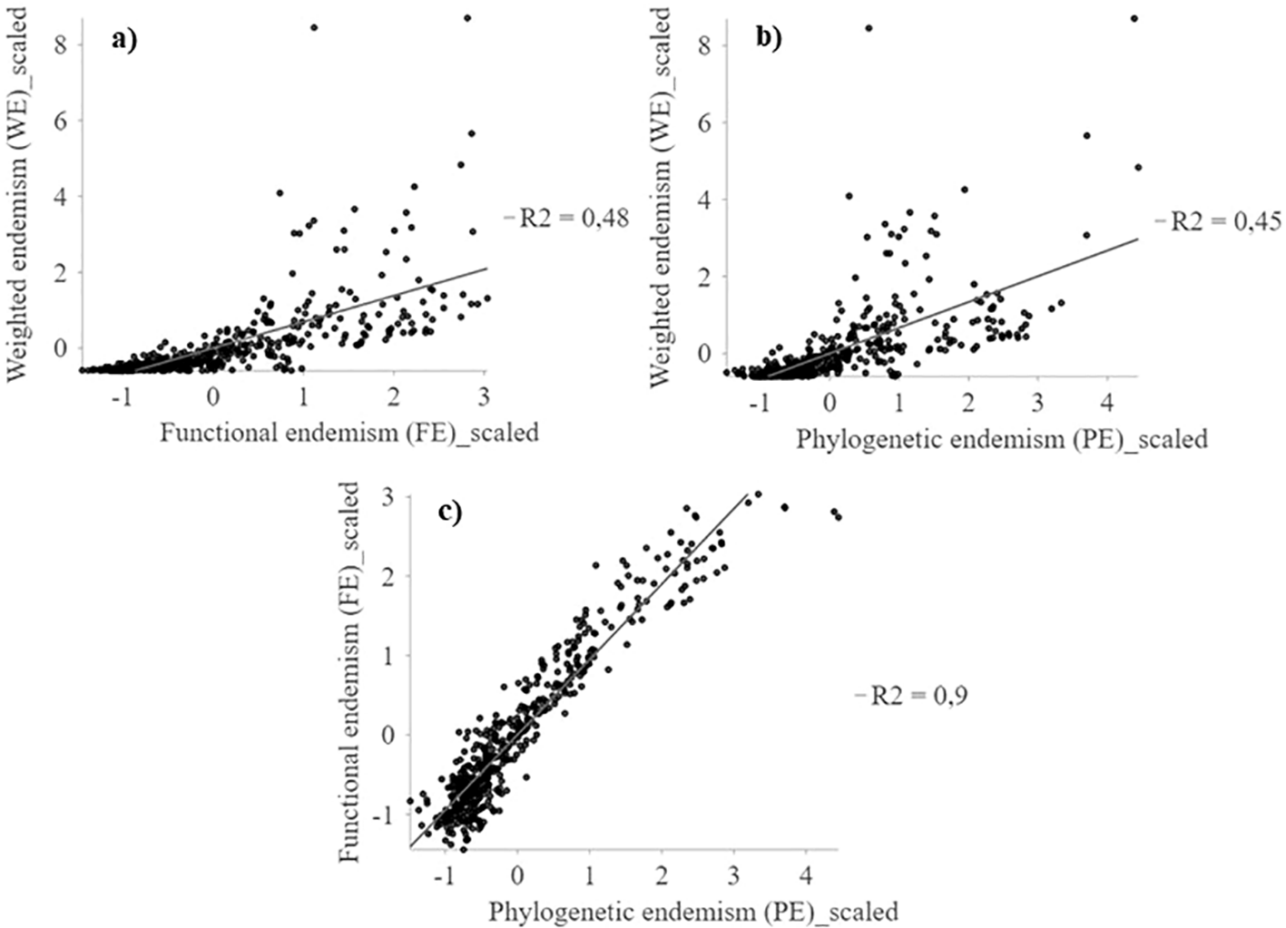
Multiscale geographically weighted regression (MGWR) between WE, FE and PE spatial patterns of the perennial flora of northern Chile. **(A)** WE~FE; **(B)** WE~PE, and **(C)** FE~PE.

**Figure 5:**
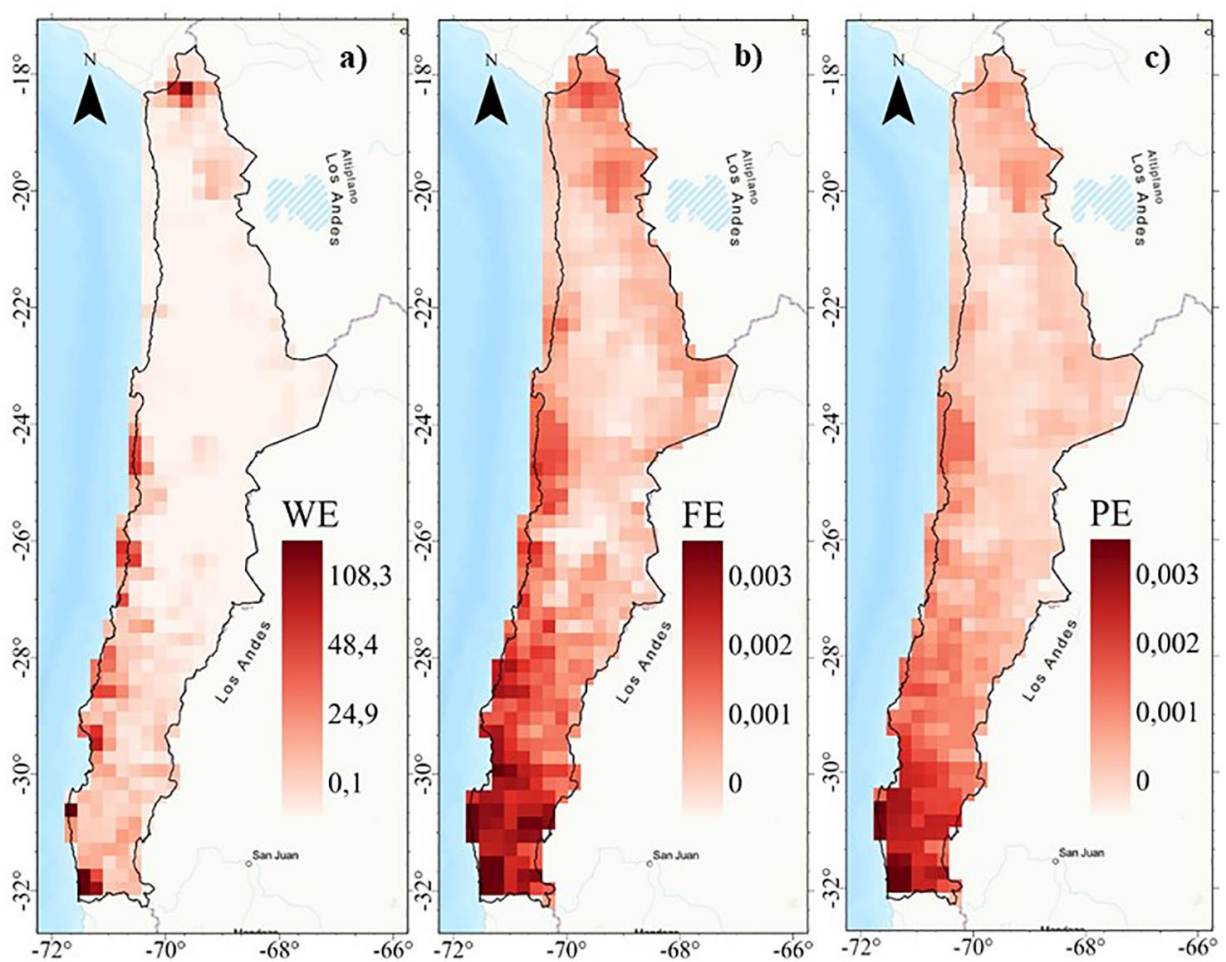
Spatial patterns of endemism: **(A)** Weighted endemism (WE), **(B)** Functional endemism (FE), and **(C)** phylogenetic endemism (PE) of the perennial flora of northern Chile.

Weighted endemism (WE) show a low association with functional endemism (FE) and phylogenetic endemism (PE) (r^2^ < 0.48) (**Figure 4**). Weighted endemism (WE) show patterns with peaks in the highlands of northern Chile (18°S) and coastal sectors between 24° and 32°S (**Figure 5**). FE and PE show a higher spatial association (r^2^ > 0.90) (**Figure 4**), concentrated in the 24°S surthem in the coastal fog oases, as well as in transition zones and northern central Chile (**Figure 5** and **Supplementary Figure S8**). The highest WE in a single cell was 108, while FE reached a maximum of 0.003 and PE was 0.003. Importantly, the spatial patterns of WE, FE, and PE are consistent regardless of spatial resolution (e.g., 100 and 75 km grids) (**Supplementary Figure S9**).

### 3.3 Spatial congruence and mismatch of diversity and endemism

There is a wide variation in MGWR (local R^2^), both spatially and numerically, among different diversity dimensions (**Supplementary Figure S10**). For TD ~ FD and FD ~ PD, areas of highest association (local R^2^ > 0.80) occur in northern Chile (18-19°S) and at different altitudes in the transitional semi-arid and pluvial-seasonal zones (26-30°S). However, areas of lower association (local R^2^≈0.50) are observed (**Supplementary Figure S10**), located in the desert, fog oasis, and northern central Chile, between 21-32°S at different elevations. TD-PD patterns show highly significant spatial association (local R^2^ > 0.88) in different areas of northern Chile (18-21°S), coastal, desert, and transitional sectors between 26- 29°S at different elevations (**Supplementary Figure S10B**).

**Figure 6:**
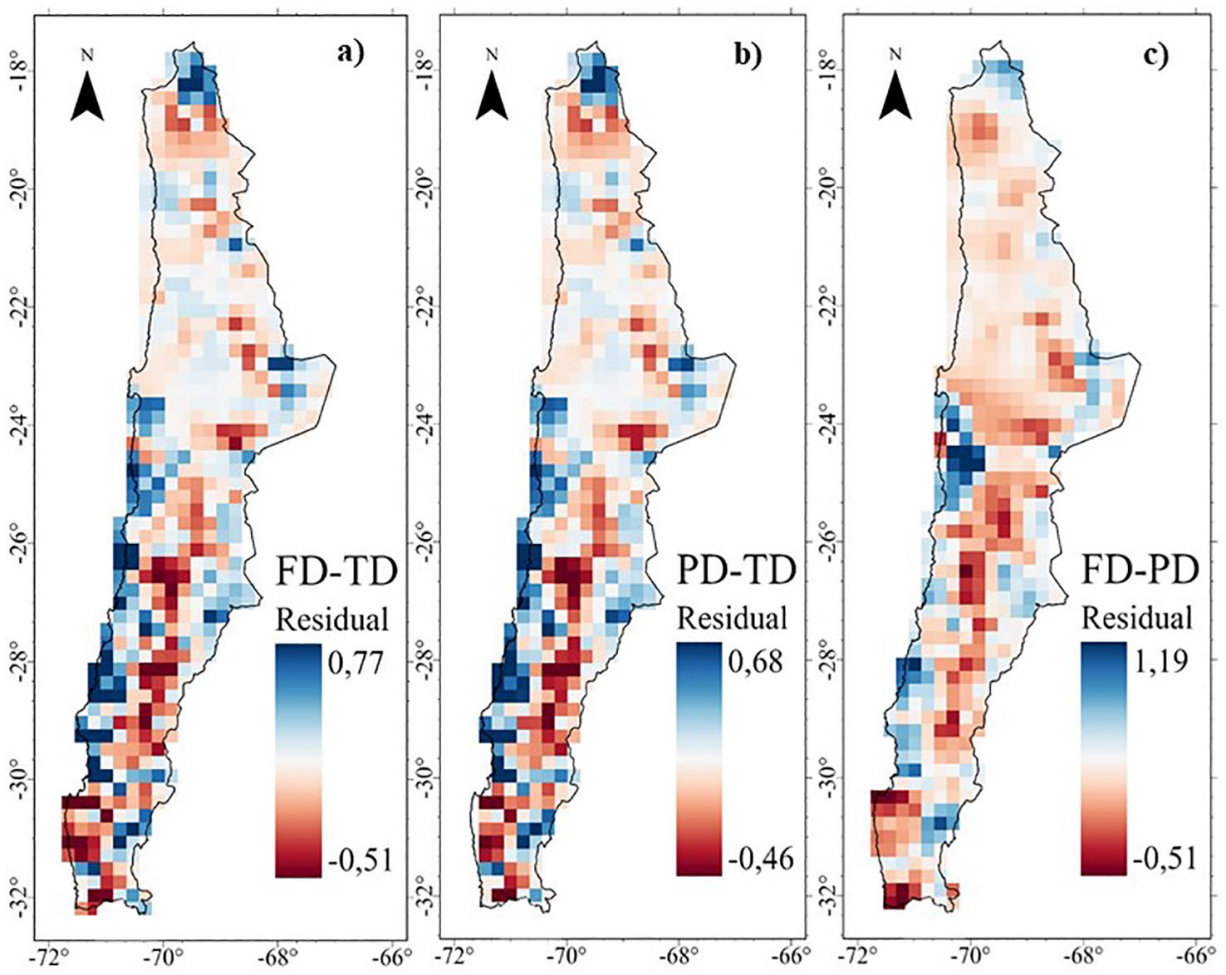
Residuals of the Multiscale Geographically Weighted Regression (MGWR) of the diversity of the perennial flora of northern Chile: **(A)** Functional - Taxonomic, **(B)** Phylogenetic - Taxonomic, and **(C)** Functional -Phylogenetic.

The MGWR residual maps show discrepancies between different diversity dimensions (**Figure 6**). For FD ~ TD and PD ~ TD, high positive residuals indicate more FD or PD than expected given TD (i.e. FD>TD and PD>TD, blue color), primarily in areas of higher water availability such as the Puna, high Andean areas and fog oases (**Figure 6**). On the other hand, negative residuals indicate less FD or PD than expected given TD (i.e., FD<TD and PD<TD, red color), which are located in desert and transitional zones (18-32°S) at different altitudes, as well as in high Andean zones influenced by the Atacama Desert (22-23°S) (**Figure 6**). High negative residuals indicate more PD than expected given the FD (i.e., PD>FD, red colors), which are located in restrictive environmental conditions such as desert areas and transition zones (18-32°S) at different altitudes (**Figure 6**).

In terms of endemism patterns, FE ~ PE shows specific areas of higher spatial association (local R^2^ > 0.50), concentrated throughout the study area, with the exception of the High Andean zone between 23-27°S (**Supplementary Figure S11**). In contrast, weighted endemism (WE) shows low association with FE and PE (local R^2^ < 0.37 and 0.36, respectively) in desert, high Andean and transitional zones (18-32°S) at different elevations (**Supplementary Figure S11**).

**Figure 7:**
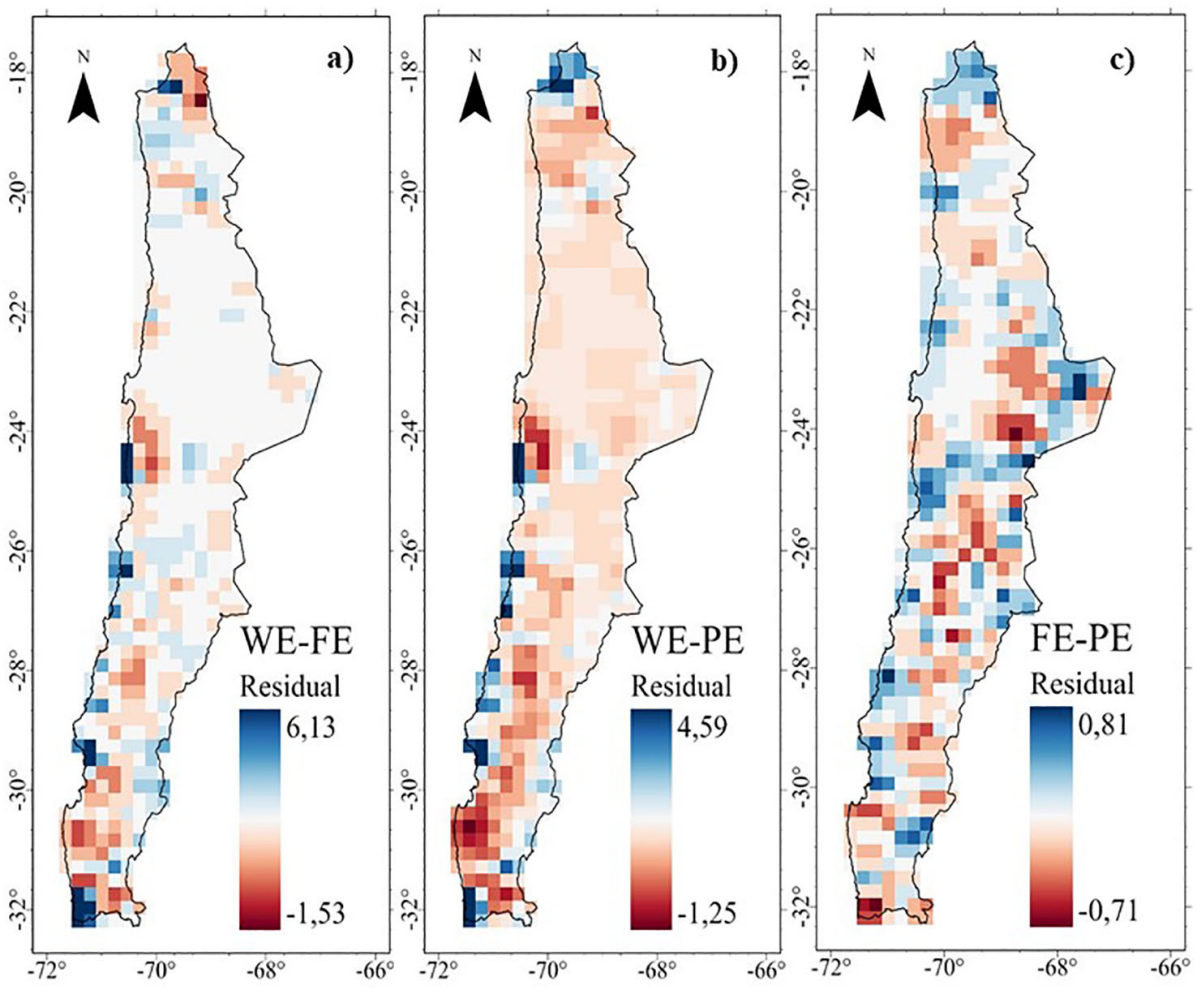
Residuals of the Multiscale Geographically Weighted Regression (MGWR) of the endemism of the perennial flora of northern Chile: **(A)** Taxonomic - Functional, **(B)** Taxonomic - Phylogenetic, and **(C)** Functional -Phylogenetic.

The MGWR residual maps show the discrepancies between the different dimensions of endemism (**Figure 7**). For WE ~ FE and WE~ PE, high positive values indicate more WE than expected given the FE and PE (i.e. WE>FE and WE>PE, blue color) in areas in the Puna (18°S) and along coastal areas (Fog oasis). In contrast, the locations where FE and PE are higher than expected given the WE (negative residuals, i.e. FE>WE and PE>WE, red color) are found in the desert, high Andean and transitional zones at different elevations. The spatial pattern for PE ~ WE is similar to that for FE ~ WE. The mismatch between PE and FE is unclear (**Figure 7**).

## 4 Discussion

### 4.1 Composition of the perennial flora of the arid and semi-arid zone of northern Chile

We found 851 perennial species inhabiting the arid and semi-arid zone of northern Chile (**Supplementary Table S1**). This number represents 15.6% of the total vascular flora of the country (5,471 species according to Rodriguez et al. (2018)), inhabiting 39.9% of the national territory. The 10 most important families by number of species represent more than 70% of the total number of species recorded. In particular, Asteraceae and Cactaceae together account for more than 43% of the species recorded (**Table 2**), whose high representation in arid and semi-arid zones has been previously reported (Guerrero et al., 2011; Moreira-Muñoz et al., 2012; Vanderplank et al., 2014). However, the hyperdominance of a small number of families that form communities is not unique to the arid and semi-arid perennial flora of Chile, as it has been documented in other ecosystems, such as Neotropical rainforests (Carim et al., 2015) and Neotropical dry forests (Banda-R et al., 2016).

### 4.2 Spatial patterns of diversity and endemism

The geographic distribution patterns of taxonomic diversity (TD), functional diversity (FD), and phylogenetic diversity (PD) in the perennial flora of northern Chile show consistent spatial correlations, as theoretically expected (De Bello et al., 2006; De Bello, 2012; Rocha-Santos et al., 2023). In particular, diversity shows a bimodal latitudinal pattern, with the highest TD, FD, and PD occurring in the Puna or highlands of northern Chile (18°S), as well as in areas south of 24°S (fog oasis, pluvial-seasonal transition zones, and northern central Chile) (**Figure 3** and **Supplementary Figure S6**). In general, these regions of high diversity correspond to areas with higher rainfall or available to plants, such as the Puna (18°S); this area benefits from the Bolivian winter edge effect, resulting in increased annual precipitation levels, primarily exceeding 350 mm (Romero and Kampf, 2003); the coastal Oasis Fog (18-31°S), known as “camanchaca”, which occurs when cold, moist air from the Pacific Ocean meets warmer land, causing the moisture to condense and form fog; and the climate of northern central of Chile with winter rainy season conditions (>300 mm) (Garreaud et al., 2003). Conversely, it is noteworthy that floristic formations with reduced TD, FD, and PD are predominantly located within the Arid Diagonal (23-29°S). This has created a severe environmental filter that allows only lineages with specific morphological traits to thrive and reproduce in the desert.

The geographic pattern of WE shows a low association with functional (FE) and phylogenetic (PE) endemism, while FE and PE patterns are more spatially aligned (**Figures 4**, **5**). The low association between the taxonomic and evolutionary dimensions of endemism has been reported in other ecosystems and spatial scales (Daru et al., 2015). The highest values of species with restricted distributions (WE) are concentrated in the northern highlands or arid Puna (18°S), documented as a center of regional plant endemism (Lambrinos et al., 2006), and the coastal Oasis Fog (24-31°S), generating local endemism hotspots in arid zones precipitation and topographic complexity (Arroyo et al., 2006; Larraín-Barrios et al., 2018).

### 4.3 Spatial discrepancies between diversity and endemism dimensions

While there is a general positive correlation between the three dimensions of diversity (taxonomic, phylogenetic, and functional), local discrepancies in these relationships (local R²) highlight the presence of different ecological and historical determinants. Lower FD than expected given TD in absolute desert, as well as in different locations of transitional semi-arid environments in north-central Chile between 24 and 32°S (**Figure 6A**), is consistent with *environmental filtering* driving similarity or convergence in morphological traits (Echeverría-Londoño et al., 2018; Larraín-Barrios et al., 2018).

According to the ecological opportunity hypothesis, we predicted lower PD than TD and FD to occur in areas of high *in situ* speciation and/or low immigration or high phenotypic differentiation driven by large ecological space (**Table 1**). The present results indicate that lower PD values than expected, given TD, are found in the desert (**Figure 6B**). Scherson et al. (2017) show that desert areas in Chile, California, and Australia have a lower PD than other vegetation types, mainly due to the relatively recent origin of modern deserts from the Oligocene to the Miocene (35 to 5 million years ago). The results also indicate a higher FD than expected given the PD along the Puna between 18 and 23°S in discontinuous areas (**Figure 6C**), consistent with an increase in growth form diversity associated with habitat heterogeneity (Lambrinos et al., 2006).

A third spatial pattern of positive FD ~ PD residuals (FD>PD) occurs in scattered areas distributed along the fog oases between 21 and 31°S (**Figure 6C**). Stotz et al. (2021) observed that fog vegetation communities between 23 and 32°S, which are affected by reduced precipitation, have lower species diversity. However, this did not translate into a loss of functional diversity, suggesting that fog oasis communities are resilient to environmental change and could therefore maintain their functional diversity.

Under the hypothesis of environmental heterogeneity, it was predicted that significant spatial variation in water and solar energy availability would generate a greater diversity of niches that species with different environmental preferences and ecological strategies can occupy (**Table 1**). The present results show that sites with higher FD than expected given TD are located in the Puna (18-24°S), as well as in scattered north-central high Andean areas between 28-32°S, and also in the fog oases (24-32°S) (**Figure 6A**), areas where desert conditions are more attenuated due to increased precipitation or fog capture (Garreaud et al., 2009). The perennial flora shows higher functional diversity than expected, possibly due to both a larger niche space and a wide range of functional strategies.

These results show a high spatial association between FE and PE (local R^2^ >0.90, **Figure 4** and **Supplementary Figure S11C**), with a higher fit in the desert and different zones in the fog oases, high Andes, and north central Chile. However, high positive values indicate more WE than expected given the FE and PE (i.e. WE>FE and WE>PE, blue color) in the coastal desert, especially in the fog oases between 24 and 32°S (**Figure 7**). In these areas, which occur as fragments of vegetation along the coastal zone, like islands in the desert above the Coastal Cordillera, they are characterized by a high diversity and endemism (Cavieres et al., 2002; Dillon, 2005). Another important factor is the coastal topography, which in some of its areas presents isolated mountains that intercept the clouds, where foggy areas develop with a layer of stratification concentrated towards the slopes (Dillon and Hoffmann-J, 1997). The topographical complexity and the presence of coastal fog are the key to the great diversity of the coastal desert flora, where the formation of local endemism’s is generated by the general isolation of the flora and the limitation of vegetation to ravines and foggy areas separated from each other (Larraín-Barrios, 2007).

## 5 Conclusion

We found evidence for geographic discrepancies, suggesting different eco-evolutionary drivers between the dimensions of perennial flora diversity and endemism across the aridity gradient in Chile. Our primary results show a strong linear covariation between the three dimensions of α-diversity (TD, PD, and FD) (r^2^>0.93). Locally, the different dimensions of α-diversity show areas of low association or spatial mismatch. They are mainly concentrated in the following areas: 1) Arid zones in the northern regions of the country (18-26°S) with lower FD or PD than expected given the TD, suggesting communities with associated *in situ* speciation processes, as well as a limitation of morpho-functional trait diversity in response to extreme environmental conditions (environmental filter hypothesis); 2) Fog oasis, between 24 and 32°S, with higher FD or PD than expected given the TD, which could be related to a variation in the availability of water and solar energy, generating a greater diversity of niches that can be occupied by species with different environmental preferences and ecological strategies (environmental heterogeneity hypothesis) and 3) In the northern Chilean Andes, especially at 18°S, with higher FD than expected given the PD, consistent with an increase in trait diversity associated with the habitat heterogeneity hypothesis.

In contrast, taxonomic endemism or WE shows a weak association with PE and FE (r2<0.48) at the regional scale, where FE and PE values are higher than expected given WE and are found in arid, high Andean and transitional zones, at different altitudes. This would indicate a greater presence of phylogenetic lineages and species with morpho-functional traits related to extreme environmental conditions and transitional biomes (arid-semiarid).

Finally, the present approach to functional diversity based on morphological traits may not be sufficient to understand the environmental and ecophysiological constraints to which plants are exposed in arid and semi-arid zones. However, the results obtained here could be used as a first approximation to contrast the use of physiological traits related to drought tolerance, as well as to compare them with finer spatial resolutions along the arid gradient of Chile.

## Data Availability

The original contributions presented in the study are included in the article/**Supplementary Material**, further inquiries can be directed to the corresponding authors.

## References

[B1] Arroyo M. T. K. Marquet P. Marticorena C. Cavieres L. Squeo F. Simonetti Zambelli J.. (2006). “Hotspot chileno, prioridad mundial para la conservación Diversidad de ecosistemas, ecosistemas terrestres,” in CONAMA. Biodiversidad de Chile: patrimonios y desafíos, Santiago de Chile: CONAMA, pp. 94–97. Available at: http://repositorio.uChile.cl/handle/2250/120068.

[B2] Arroyo M. T. K. Squeo F. A. Armesto J. J. Villagran C. (1988). Effects of aridity on plant diversity in the northern Chilean Andes: results of a natural experiment. Ann. Missouri Botanical Garden 75, 55–78. doi: 10.2307/2399466

[B3] Arroyo M. T. K. Castor C. Marticorena C. Muñoz M. Cavieres L. Matthei O.. (1998). The flora of Llullaillaco National Park located in the transitional winter-summer rainfall area of the northern Chilean Andes. Gayana Botánica 55, 93–110.

[B4] Banda-R K. Delgado-Salinas A. Dexter K. G. Linares-Palomino R. Oliveira-Filho A. Prado D.. (2016). Plant diversity patterns in neotropical dry forests and their conservation implications. Science 353, 1383–1387. doi: 10.1126/science.aaf5080 27708031

[B5] Bull-Hereñu K. Martínez E. A. Squeo F. A. (2005). Structure and genetic diversity in *Colliguaja odorifera* Mol.(Euphorbiaceae), a shrub subjected to Pleisto-Holocenic natural perturbations in a mediterranean South American region. J. Biogeo. 32, 1129–1138. doi: 10.1111/j.1365-2699.2004.01209.x

[B6] Cadotte M. W. Carscadden K. Mirotchnick N. (2011). Beyond species: functional diversity and the maintenance of ecological processes and services. J. Appl. Ecol. 48, 1079–1087. doi: 10.1111/j.1365-2664.2011.02048.x

[B7] Cai L. Kreft H. Taylor A. Denelle P. Schrader J. Essl F.. (2023). Global models and predictions of plant diversity based on advanced machine learning techniques. New Phytol. 237, 1432–1445. doi: 10.1111/nph.18533 36375492

[B8] Carim M. D. J. V. Da Silva Guimarães J. R. Tostes L. D. C. L. Takiyama L. R. Wittmann F. (2015). Composition, structure and floristic diversity in dense rain forest in the Eastern Amazon, Amapá, Brazil. Acta Scientiarum. Biol. Sci. 37, 419–426. doi: 10.4025/actascibiolsci.v37i4.27536

[B9] Cavieres L. A. Arroyo M. T. Posadas P. Marticorena C. Matthei O. Rodríguez R.. (2002). Identification of priority areas for conservation in an arid zone: application of parsimony analysis of endemicity in the vascular flora of the Antofagasta region, northern Chile. Biodivers. Conserv. 11, 1301–1311. doi: 10.1023/A:1016001714358

[B10] Chesson P. (2000). General theory of competitive coexistence in spatially varying environments. Theor. Popul Biol. 58, 211–237. doi: 10.1006/tpbi.2000.1486 11120650

[B11] Daru B. H. Van Der Bank M. Davies T. J. (2015). Spatial incongruence among hotspots and complementary areas of tree diversity in southern Africa. Diversity Distribut. 21, 769–780. doi: 10.1111/ddi.12290

[B12] Davies T. J. Buckley L. B. (2011). Phylogenetic diversity as a window into the evolutionary and biogeographic histories of present-day richness gradients for mammals. Philos. Trans. R. Soc. B: Biol. Sci. 366, 2414–2425. doi: 10.1098/rstb.2011.0058 PMC3130431 21768156

[B13] De Bello F. (2012). The quest for trait convergence and divergence in community assembly: Are null-models the magic wand? Global Ecol. Biogeogry 21, 312–317. doi: 10.1111/j.1466-8238.2011.00682.x

[B14] De Bello F. Lepš J. Sebastià M. T. (2006). Variations in species and functional plant diversity along climatic and grazing gradients. Ecography 29, 801–810. doi: 10.1111/j.2006.0906-7590.04683.x

[B15] Dillon M. (2005). “Solanaceae of the lomas formations of coastal Peru and Chile,” in A festschrift for william G. D’Arcy: the legacy of a taxonomist. Eds. Hollowell V. Keating T. Lewis W. Croat T. (Monographs in Systematic Botany from the Missouri Botanical Garden) 104, 131–155.

[B16] Dillon M. O. Hoffmann-J A. E. (1997). “Lomas formations of the atacama desert northern Chile,” in Centres of Plant Diversity: A guide and Strategy for their Conservation. Eds. Davis S. D. Heywood V. H. Herrera-MacBryde O. Villa-Lobos J. Hamilton A. C. (WWF, IUCN, Oxford, U.K).

[B17] Doxa A. Devictor V. Baumel A. Pavon D. Médail F. Leriche A. (2020). Beyond taxonomic diversity: Revealing spatial mismatches in phylogenetic and functional diversity facets in Mediterranean tree communities in southern France. For. Ecol. Manage. 474, 118318. doi: 10.1016/j.foreco.2020.118318

[B18] Echeverría-Londoño S. Enquist B. J. Neves D. M. Violle C. Boyle B. Kraft N. J. B.. (2018). Plant functional diversity and the biogeography of biomes in North and South America. Front. Ecol. Evol. 6. doi: 10.3389/fevo.2018.00219

[B19] ESRI. (2023). ArcgisPro 3.1.2 (Redlands, CA: Environmental Systems Research Institute).

[B20] Farr T. G. Rosen P. A. Caro E. Crippen R. Duren R. Hensley S. (2007). The shuttle radar topography mission. Rev Geophys. 45. doi: 10.1029/2005RG000183

[B21] Faith D. P. (1992). Conservation evaluation and phylogenetic diversity. Biol. Conserv. 61, 1–10. doi: 10.1016/0006-3207(92)91201-3

[B22] Fick S. E. Hijmans R. J. (2017). WorldClim 2: new 1‐km spatial resolution climate surfaces for global land areas. Int J Climatol. 37, 4302–4315. doi: 10.1002/joc.5086

[B23] Fotheringham A. S. Yang W. Kang W. (2017). Multiscale geographically weighted regression (MGWR). Ann. Am. Assoc. Geographers 107, 1247–1265. doi: 10.1080/24694452.2017.1352480

[B24] Freitas C. Brum F. T. Cássia-Silva C. Maracahipes L. Carlucci M. B. Collevatti R. G.. (2021). Incongruent spatial distribution of taxonomic, phylogenetic, and functional diversity in neotropical cocosoid palms. Front. Forests Global Change 4, 739468. doi: 10.3389/ffgc.2021.739468

[B25] Garcillán P. P. Ezcurra E. Riemann H. (2003). Distribution and species richness of woody dryland legumes in Baja California, Mexico. J. Veg. Sci. 14, 475–486. doi: 10.1111/j.1654-1103.2003.tb02174.x

[B26] Garreaud R. Vuille M. Clement A. (2003). The climate of the Altiplano: Observed current conditions and past change mechanisms. Palaeogeo Palaeoclimatol. Palaeoecol. 194, 5–22. doi: 10.1016/S0031-0182(03)00269-4

[B27] Garreaud R. D. Vuille M. Compagnucci R. Marengo J. (2009). Present-day south american climate. Palaeogeo Palaeoclimatol. Palaeoecol. 281, 180–195. doi: 10.1016/j.palaeo.2007.10.032

[B28] Gower J. C. (1966). Some distance properties of latent root and vector methods used in multivariate analysis. Biometrika 53, 325–338. doi: 10.1093/biomet/53.3-4.325

[B29] Grime J. P. (2006). Trait convergence and trait divergence in herbaceous plant communities: mechanisms and consequences. J. Vegetation Sci. 17. 2, 255–260. doi: 10.1111/j.1654-1103.2006.tb02444.x

[B30] Guerrero P. C. Durán A. P. Walter H. E. (2011). Latitudinal and altitudinal patterns of the endemic cacti from the Atacama Desert to Mediterranean Chile. J. Arid Environments 75, 991–997. doi: 10.1016/j.jaridenv.2011.04.036

[B31] Hawkins B. A. (2001). Ecology’s oldest pattern? Trends Ecol. Evol. 16, 470. doi: 10.1016/s0160-9327(00)01369-7

[B32] Hijmans R. (2024). Terra: spatial data analysis. Available online at: https://cran.r-project.org/web/packages/terra/terra.pdf. (Accessed January 14, 2024).

[B33] Hughes C. Eastwood R. (2006). Island radiation on a continental scale: exceptional rates of plant diversification after uplift of the Andes. Proc. Natl. Acad. Sci. 103, 10334–10339. doi: 10.1073/pnas.0601928103 16801546 PMC1502458

[B34] Isaac N. J. Turvey S. T. Collen B. Waterman C. Baillie J. E. (2007). Mammals on the EDGE: conservation priorities based on threat and phylogeny. PloS One 2, e296. doi: 10.1371/journal.pone.0000296 17375184 PMC1808424

[B35] Jin Y. Qian H. (2023). U. PhyloMaker: An R package that can generate large phylogenetic trees for plants and animals. Plant Diversity 45, 347–352. doi: 10.1016/j.pld.2022.12.007 37397595 PMC10311187

[B36] Karger D. Conrad O. Böhner J. Kawohl T. Kreft H. Soria-Auza R. W.. (2017). Climatologies at high resolution for the Earth land surface areas. Sci. Data 4, 170122. doi: 10.1038/sdata.2017.122 28872642 PMC5584396

[B37] Keddy P. A. (1992). Assembly and response rules: two goals for predictive community ecology. J. vegetation Sci. 3, 157–164. doi: 10.2307/3235676

[B38] Keppel G. Van Niel K. P. Wardell-Johnson G. W. Yates C. J. Byrne M. Mucina L.. (2012). Refugia: identifying and understanding safe havens for biodiversity under climate change. Global Ecol. Biogeo. 21, 393–404. doi: 10.1111/j.1466-8238.2011.00686.x

[B39] Kreft H. Jetz W. (2007). Global patterns and determinants of vascular plant diversity. Proc. Natl. Acad. Sci. 104, 5925–5930. doi: 10.1073/pnas.0608361104 17379667 PMC1851593

[B40] Laffan S. W. Crisp M. D. (2003). Assessing endemism at multiple spatial scales, with an example from the Australian vascular flora. J. Biogeogr. 30, 511–520. doi: 10.1046/j.1365-2699.2003.00875.x

[B41] Laffan S. W. Lubarsky E. Rosauer Df. (2008). Biodiverse, a spatial analysis tool to analyse species (and other) diversity (Sydney, Australia: School of Biology, Earth and Environmental Sciences, University of New South Wales).

[B42] Lambrinos J. G. Kleier C. C. Rundel P. W. (2006). Plant community variation across a puna landscape in the Chilean Andes. Rev. Chil. Hist. Natural 79, 233–243. doi: 10.4067/S0716-078X2006000200009

[B43] Larraín-Barrios C. (2007). “Relaciones florísticas entre oasis de neblina del desierto costero del norte de Chile,” in Tesis para optar al título de Ingeniero en Recursos Renovables (Universidad de Chile). Available online at: https://repositorio.uchile.cl/handle/2250/101884. (Accessed March 1, 2021).

[B44] Larraín-Barrios B. Faúndez-Yancas L. Búrquez A. (2018). Plant functional trait structure in two fog deserts of America. Flora 243, 1–10. doi: 10.1016/j.flora.2018.03.005

[B45] Le Bagousse-Pinguet Y. Soliveres S. Gross N. Torices R. Berdugo M. Maestre F. T. (2019). Phylogenetic, functional, and taxonomic richness have both positive and negative effects on ecosystem multifunctionality. Proc. Natl. Acad. Sci. 116, 8419–8424. doi: 10.1073/pnas.1815727116 30948639 PMC6486734

[B46] Luebert F. Fuentes-Castillo T. Pliscoff P. García N. Román M. J. Vera D.. (2022). GEOGRAPHIC patterns of vascular plant diversity and endemism using different taxonomic and spatial units. Diversity 14, 271. doi: 10.3390/d14040271

[B47] Luebert F. Gajardo R. (2000). Vegetación de los Andes áridos del norte de Chile. Lazaroa 21, 111–130.

[B48] Luebert F. Weigend M. (2014). Phylogenetic insights into Andean plant diversification. Front. Ecol. Evol. 2, 1–17. doi: 10.3389/fevo.2014.00027

[B49] Maechler M. Rousseeuw P. Struyf A. Hubert M. Hornik K. (2021). cluster: cluster analysis basics and extensions. R package version 2.1.2. https://CRAN.R-project.org/package=cluster.

[B50] Mammola S. Carmona C. P. Guillerme T. Cardoso P. (2021). Concepts and applications in functional diversity. Functional Ecology 35, 1869–1885. doi: 10.1111/1365-2435.13882

[B51] Miller A. (1976). “The climate of Chile,” in Climates of Central and South America. Ed. Schwerdtfeger W. (Amsterdam, The Netherlands: Elsevier), 113–145. doi: 10.1002/qj.49710343520

[B52] Mishler B. D. Knerr N. González-Orozco C. E. Thornhill A. H. Laffan S. W. Miller J. T. (2014). Phylogenetic measures of biodiversity and neo-and paleo-endemism in Australian Acacia. Nat. Commun. 5, 4473. doi: 10.1038/ncomms5473 25034856

[B53] Moreira-Muñoz A. Morales V. Muñoz-Schick M. (2012). Actualización sistemática y distribución geográfica de Mutisioideae (Asteraceae) de Chile. Gayana Botánica 69, 9–29. doi: 10.4067/S0717-66432012000100003

[B54] Mráz P. Barabas D. Lengyelová L. Turis P. Schmotzer A. Janišová M.. (2016). Vascular plant endemism in the Western Carpathians: spatial patterns, environmental correlates and taxon traits. Biol. J. Linn. Soc 119, 1095–8312. doi: 10.1111/bij.12792

[B55] Newbery D. Campbell E. Proctor J. Still M. J. (1996). Primary lowland dipterocarp forest at Danum Valley, Sabah, Malaysia. Species composition and patterns in the understorey. Vegetatio 122, 193–220. doi: 10.1007/BF00044700

[B56] Ochoa-Ochoa L. M. Mejía-Domínguez N. R. Velasco J. A. Dimitrov D. Marske K. A. (2020). Dimensions of amphibian alpha diversity in the New World. J. Biogeo. 47, 2293–2302. doi: 10.1111/jbi.13948

[B57] Oliveira U. Soares-Filho B. Leitão R. F. M. Rodrigues H. O. (2019). BioDinamica: a toolkit for analyses of biodiversity and biogeography on the Dinamica-EGO modelling platform. Peerj 7, e7213–e7213. doi: 10.7717/peerj.7213 31338256 PMC6628879

[B58] Pebesma E. Bivand R. (2023). Spatial data science: with applications in R. 1st ed. (New York: Chapman and Hall/CRC), 314. doi: 10.1201/9780429459016

[B59] Pérez-Escobar O. A. Zizka A. Bermúdez M. A. Meseguer A. S. Condamine F. L. Hoorn C.. (2022). The Andes through time: evolution and distribution of Andean floras. Trends Plant Sci. 27, 364–378. doi: 10.1016/j.tplants.2021.09.010 35000859

[B60] Petchey O. L. Gaston K. J. (2006). Functional diversity: back to basics and looking forward. Ecol. Lett. 9, 741–758. doi: 10.1111/j.1461-0248.2006.00924.x 16706917

[B61] Qian H. Jin Y. (2016). An updated megaphylogeny of plants, a tool for generating plant phylogenies and an analysis of phylogenetic community structure. J. Plant Ecol. 9, 233–239. doi: 10.1093/jpe/rtv047

[B62] Qian H. Kessler M. Deng T. Jin Y. (2021). Patterns and drivers of phylogenetic structure of pteridophytes in China. Global Ecol. Biogeo. 30, 1835–1846. doi: 10.1111/geb.13349

[B63] Qian H. Qian S. Zhang J. Kessler M. (2024). Effects of climate and environmental heterogeneity on the phylogenetic structure of regional angiosperm floras worldwide. Nat. Commun. 15, 1079. doi: 10.1038/s41467-024-45155-9 38316752 PMC10844608

[B64] Rocha-Santos L. Faria D. Mariano-Neto E. Andrade E. R. Bomfim J. A. Talora D. C.. (2023). Taxonomic, phylogenetic and functional responses of plant communities in different life-stages to forest cover loss. Perspect. Ecol. Conserv. 21, 136–142. doi: 10.1016/j.pecon.2023.03.001

[B65] Rodriguez R. Marticorena C. Alarcón D. Baeza C. Cavieres L. Finot V.. (2018). Catálogo de las plantas vasculares de Chile. Gayana. Botánica 75, 1–430. doi: 10.4067/S0717-66432018000100001

[B66] Romero H. Kampf S. (2003). “Impacts of climate fluctuations and climate changes on the sustainable development of the arid Norte Grande in Chile,” in Climate and water: transboundary challenges in the Americas (Springer Netherlands, Dordrecht), 83–115.

[B67] Rosauer D. Laffan S. W. Crisp M. D. Donnellan S. C. Cook L. G. (2009). Phylogenetic endemism: A new approach for identifying geographical concentrations of evolutionary history. Mol. Ecol. 18, 4061–4072. doi: 10.1111/j.1365-294X.2009.04311.x 19754516

[B68] Rundel P. Dillon M. Palma B. Mooney H. Gulmon S. Ehleringer J. R. (1991). The phytogeography and ecology of the coastal Atacama and Peruvian deserts. Aliso 13, 1–494. doi: 10.5642/aliso

[B69] Rundel P. W. Gibson A. C. Midgley G. S. Wand S. J. E. Palma B. Kleier C.. (2002). Ecological and ecophysiological patterns in a pre-altiplano shrubland of the Andean Cordillera in northern Chile. Plant Ecol. 169, 179–193. doi: 10.1023/A:1026075721045

[B70] Sarricolea P. Romero H. (2015). Variabilidad y cambios climáticos observados y esperados en el Altiplano del norte de Chile. Rev. geografía Norte Grande 62, 169–183. doi: 10.4067/S0718-34022015000300010

[B71] Schall J. J. Pianka E. R. (1978). Geographical trends in numbers of species. Science 201, 679–686. doi: 10.1126/science.201.4357.679 17750221

[B72] Scherson R. A. Thornhill A. H. Urbina-Casanova R. Freyman W. A. Pliscoff P. A. Mishler B. D. (2017). Spatial phylogenetics of the vascular flora of Chile. Mol. Phylogenet. Evol. 112, 88–95. doi: 10.1016/j.ympev.2017.04.021 28450227

[B73] Simpson G. G. (1945). SECTION OF BIOLOGY: tempo and mode in evolution. Trans. New York Acad. Sci. 8, 45–60. doi: 10.1111/j.2164-0947.1945.tb00215.x 21012247

[B74] Skeels A. Yaxley K. J. (2023). Functional endemism captures hotspots of unique phenotypes and restricted ranges. Ecography 2023, e06913. doi: 10.1111/ecog.06913

[B75] Smith S. A. Brown J. W. (2018). Constructing a broadly inclusive seedplant phylogeny. Am. J. Bot. 105, 302–314. doi: 10.1002/ajb2.1019 29746720

[B76] Stotz G. C. Salgado-Luarte C. Vigil A. T. De La Cruz H. J. Pastén-Marambio V. Gianoli E. (2021). Habitat-islands in the coastal Atacama Desert: loss of functional redundancy, but not of functional diversity, with decreased precipitation. Ann. Bot. 127, 669–680. doi: 10.1093/aob/mcaa206 33515007 PMC8052923

[B77] Swenson N. G. (2011). The role of evolutionary processes in producing biodiversity patterns, and the interrelationships between taxonomic, functional and phylogenetic biodiversity. Am. J. Bot. 98, 472–480. doi: 10.3732/ajb.1000289 21613140

[B78] Taylor A. Weigelt P. Denelle P. Cai L. Kreft H. (2023). The contribution of plant life and growth forms to global gradients of vascular plant diversity. New Phytol. 240, 1548–1560. doi: 10.1111/nph.19011 37264995

[B79] Testolin R. Attorre F. Jiménez-Alfaro B. (2020). Global distribution and bioclimatic characterization of alpine biomes. Ecography 43, 779–788. doi: 10.1111/ecog.05012

[B80] Thornhill A. H. Baldwin B. G. Freyman W. A. Nosratinia S. Kling M. M. Morueta-Holme N.. (2017). Spatial phylogenetics of the native California flora. BMC Biol. 15, 96. doi: 10.1186/s12915-017-0435-x 29073895 PMC5658987

[B81] Thornhill A. H. Mishler B. D. Knerr N. J. González-Orozco C. E. Costion C. M. Crayn D. M.. (2016). Continental-scale spatial phylogenetics of Australian angiosperms provides insights into ecology, evolution and conservation. J. Biogeogr. 43, 2085–2098. doi: 10.1111/jbi.12797

[B82] Tilman D. (2001). “Functional diversity,” in Encyclopedia of biodiversity. Second Edition, vol. 3 . Ed. Levin S. A. (San Diego, CA., Elsevier Inc.: Academic Press), 109–120. doi: 10.1016/B978-0-12-384719-5.00061-7

[B83] Vanderplank S. E. Moreira-Muñoz A. Hobohm C. Pils G. Noroozi J. Clark V. R.. (2014). “Endemism in mainland regions - case studies: central Chile ecoregion,” in Endemism in vascular plants. Series plant and vegetation, vol. 9 . Ed. Hobohm C. (Springer, Dordrecht), 205–308.

[B84] Villagrán C. Kalin Arroyo M. T. Marticorena C. (1983). Efectos de la desertización en la distribución de la flora andina de Chile. Revista Chilena de Historia Natural 56, 137–157.

[B85] Volaire F. Gleason S. M. Delzon S. (2020). What do you mean “functional” in ecology? Patterns versus processes. Ecol. Evol. 10, 11875–11885. doi: 10.1002/ece3.6781 33209257 PMC7663066

[B86] Voskamp A. Baker D. J. Stephens P. A. Valdes P. J. Willis S. G. (2017). Global patterns in the divergence between phylogenetic diversity and species richness in terrestrial birds. J. Biogeogr.; 44, 709–721. doi: 10.1111/jbi.12916

[B87] Wellborn G. A. Langerhans R.B. (2015). Ecological opportunity and the adaptive diversification of lineages. Ecol. Evol. 5.1, 176–195. doi: 10.1002/ece3.1347 25628875 PMC4298445

[B88] Wickham H. François R. Henry L. Müller K. Vaughan D. (2023). Dplyr: A grammar of data manipulation. R package version 1.1.4. Available online at: https://CRAN.R-project.org/package=dplyr. (Accessed January 16, 2024).

[B89] Xu X. Dimitrov D. Shrestha N. Rahbek C. Wang Z. (2019). A consistent species richness–climate relationship for oaks across the Northern Hemisphere. Global Ecol. Biogeo. 28, 1051–1066. doi: 10.1111/geb.12913

[B90] Yue J. Li R. (2021). Phylogenetic relatedness of woody angiosperm assemblages and its environmental determinants along a subtropical elevational gradient in China. Plant Diversity 43, 111–116. doi: 10.1016/j.pld.2020.08.003 33997543 PMC8103416

[B91] Zeng Q. Reid J. Saintilan N. Colloff M. J. Lei G. Wen L. (2019). Contrasting diversity patterns of breeding Anatidae in the Northern and Southern Hemispheres. Ecol. Evol. 9, 9990–10003. doi: 10.1002/ece3.5540 31548882 PMC6746110

[B92] Zhang Y. Qian L. Spalink D. Sun L. Chen J. Sun H. (2021). Spatial phylogenetics of two topographic extremes of the Hengduan Mountains in southwestern China and its implications for biodiversity conservation. Plant Diversity 43, 181–191. doi: 10.1016/j.pld.2020.09.001 34195502 PMC8233532

[B93] Zhou Y. Wang S. Njogu A. W. Ochola A. C. Boru B. H. Mwachala G.. (2019). Spatial congruence or mismatch between phylogenetic and functional structure of seed plants along a tropical elevational gradient: different traits have different patterns. Front. Ecol. Evol. 7. doi: 10.3389/fevo.2019.00100 PMC6476750 31031922

[B94] Zobel M. (1997). The relative of species pools in determining plant species richness: an alternative explanation of species coexistence? Trends Ecol. Evol. 12, 266–269. doi: 10.1016/S0169-5347(97)01096-3 21238064

