# Supplementary material for: Spatial patterns of congruence or mismatch between taxonomic, functional, and phylogenetic diversity and endemism of perennial flora along the aridity gradient of Chile
Source: Front Plant Sci. 2024 Aug 30;15:1418673. doi: 10.3389/fpls.2024.1418673 (PMC11392779; doi:10.3389/fpls.2024.1418673)
Supplement: Supplementary file 1 [file DataSheet1.pdf]

Supplementary Material

Article title: Spatial patterns of congruence or mismatch between taxonomic, functional and phylogenetic diversity and endemism of perennial flora along the aridity gradient of Chile

Paola Poch [1,2]*, Elie Poulin [1,2] María Fernanda Pérez [3], Gioconda Peralta[3] and Luis Felipe Hinojosa [2*]
[1] Institute of Ecology and Biodiversity (IEB), Santiago, Chile.
[2] Departamento de Ciencias Ecológicas, Facultad de Ciencias, Universidad de Chile, Las Palmeras 3425, Santiago, Chile.
[3] Departamento de Ecología, Facultad de Ciencias Biológicas, Pontificia Universidad Católica de Chile, Santiago, Chile.

Correspondence: Paola Poch ppoch@ieb-chile.cl; **Luis F. Hinojosa** lfhinojosa@uchile.cl

The following Supporting Information is available for this article:

Fig. S1. Redundancy values and spatial resolutions (100, 75, 50 and 25 km grid cells).

Fig. S2. Plots of number of specimens as a function of species richness (100, 75, 50 and 25 km grid cells).

Fig. S3. Phylogenetic reconstruction for the perennial flora of the arid and semi-arid zones of Chile.

Fig. S4. UPGMA dendrogram of functional traits of the perennial flora of the arid and semiarid zones of Chile.

Fig. S5. Percent of perennial species recorded by life form in the arid and semiarid zones of Chile.

Fig. S6. Latitudinal gradient of perennial flora diversity in the aridity gradient of Chile (a) Taxonomic (TD); (b) Functional (FD); and (c) Phylogenetic (PD).

Fig. S7. Spatial patterns of diversity at different resolutions (100 and 75 km grid cells) of the perennial flora of northern Chile. a) TD with 100 km grid cell; b) FD with 100 km grid cell; c) PD with 100 km grid cell; d) TD with 75 km grid cell; e) FD with 75 km grid cell; and e) PD with 75 km grid cell.

Fig. S8. Latitudinal gradient of the perennial flora endemism in the aridity gradient of Chile (a) Taxonomic (WE); (b) Functional (FE); and (c) Phylogenetic (PE).

Fig. S9. Spatial patterns of endemism at different resolutions (100 and 75 km grid cells) of the perennial flora of northern Chile. a) WE with 100 km grid cell; b) FE with 100 km grid cell; c) PE with 100 km grid cell; d) WE with 75 km grid cell; e) FE with 75 km grid cell; and e) PE with 75 km grid cell.

Fig. S10. Multiscale Geographically Weighted Regression (MGWR) of the diversity of the perennial flora of northern Chile. The colour schemes represent the local R^2 value: a) Taxonomic~Functional, b) Taxonomic~Phylogenetic, and c) Functional~Phylogenetic.

Fig. S11. Multiscale Geographically Weighted Regression (MGWR) of the endemism of the perennial flora of northern Chile. The colour schemes represent the local R^2 value: (a) Taxonomic ~ Functional, (b) Taxonomic~Phylogenetic, and (c) Functional~Phylogenetic.

Supplementary Figures

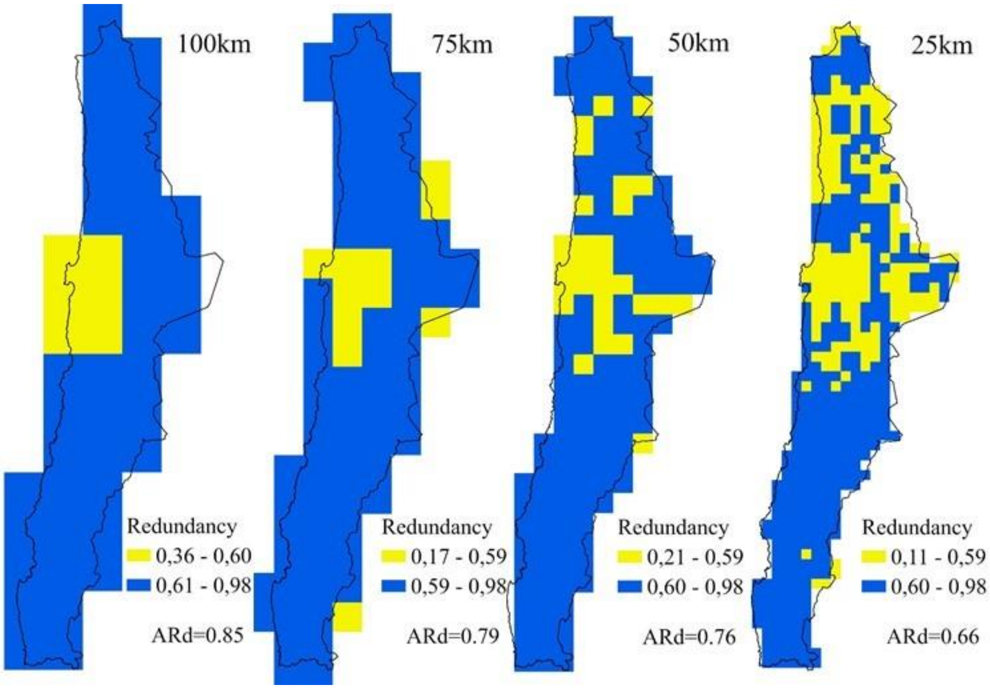

Fig. S1. Redundancy values and spatial resolutions (100, 75, 50 and 25 km grid cells).

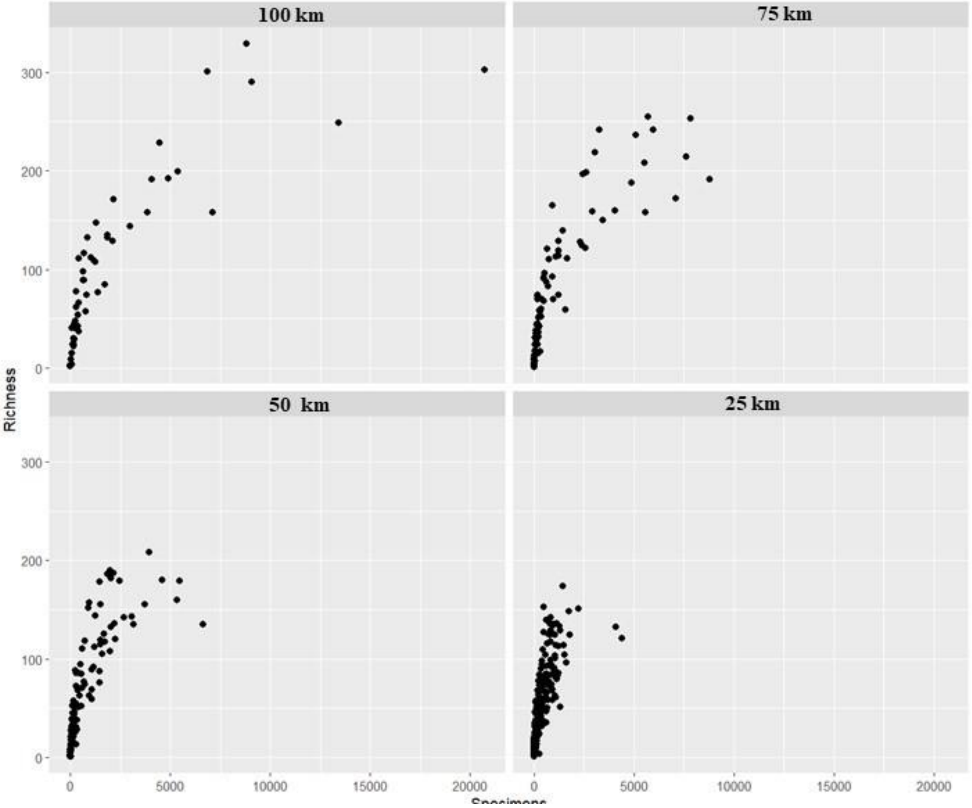

Fig. S2. Plots of number of specimens as a function of species richness (100, 75, 50 and 25 km grid cells).

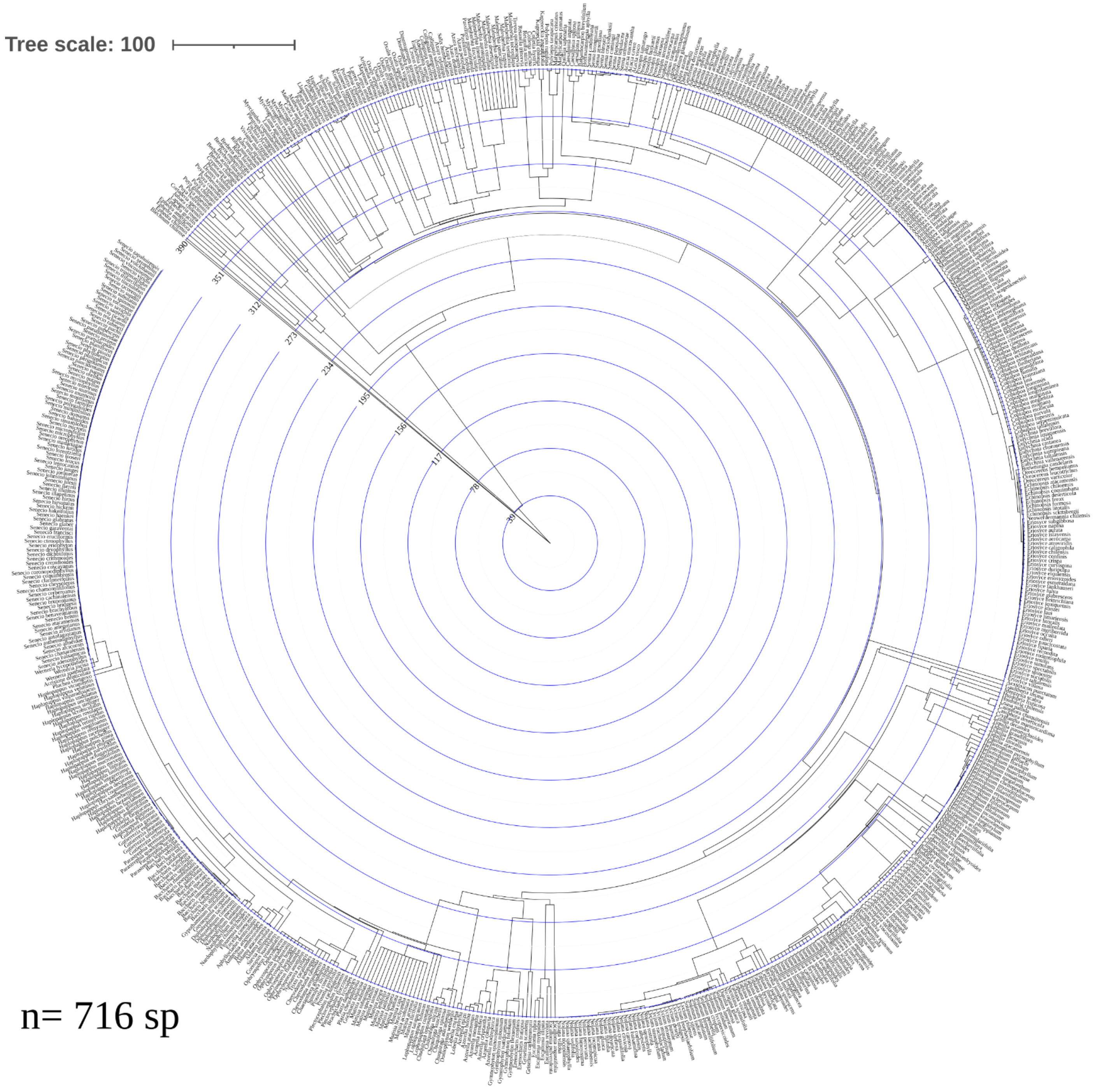

Fig. S3. Phylogenetic reconstruction for the perennial flora of the arid and semi-arid zones of Chile.

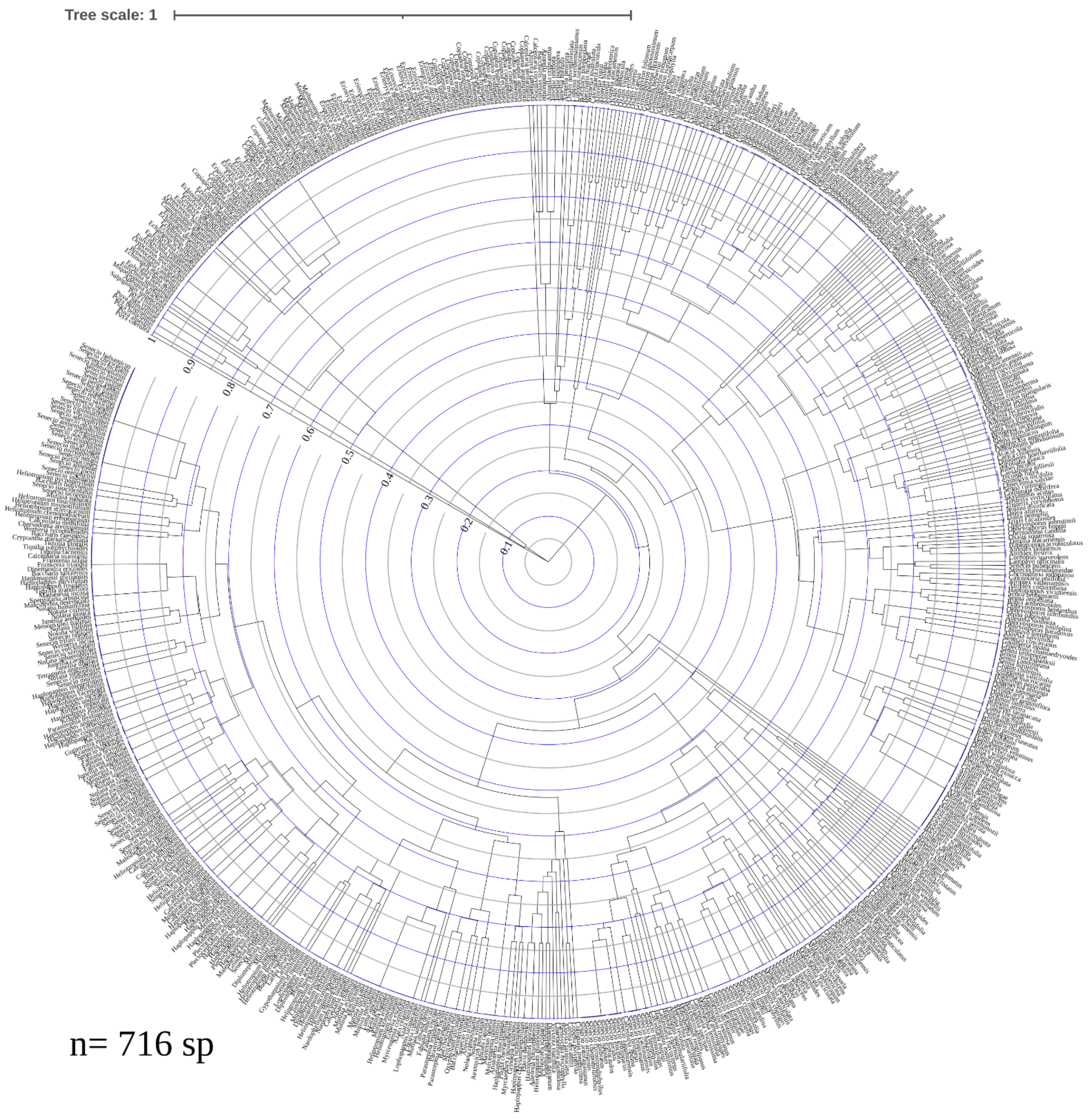

Fig. S4. UPGMA dendrogram of functional traits of the perennial flora of the arid and semiarid zones of Chile.

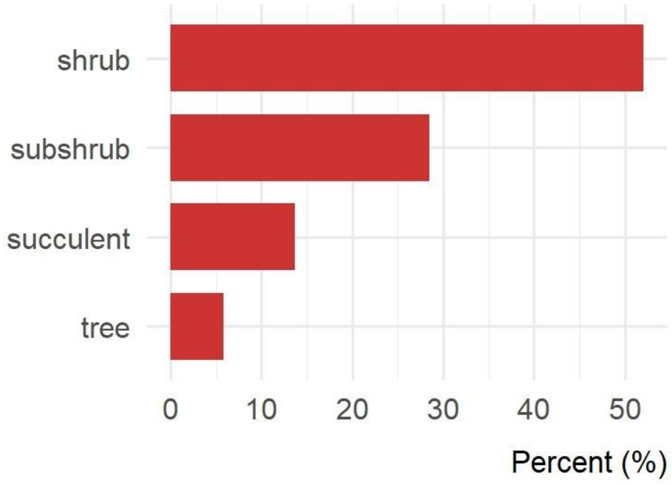

Fig. S5. Percent of perennial species recorded by life form in the arid and semiarid zones of Chile.

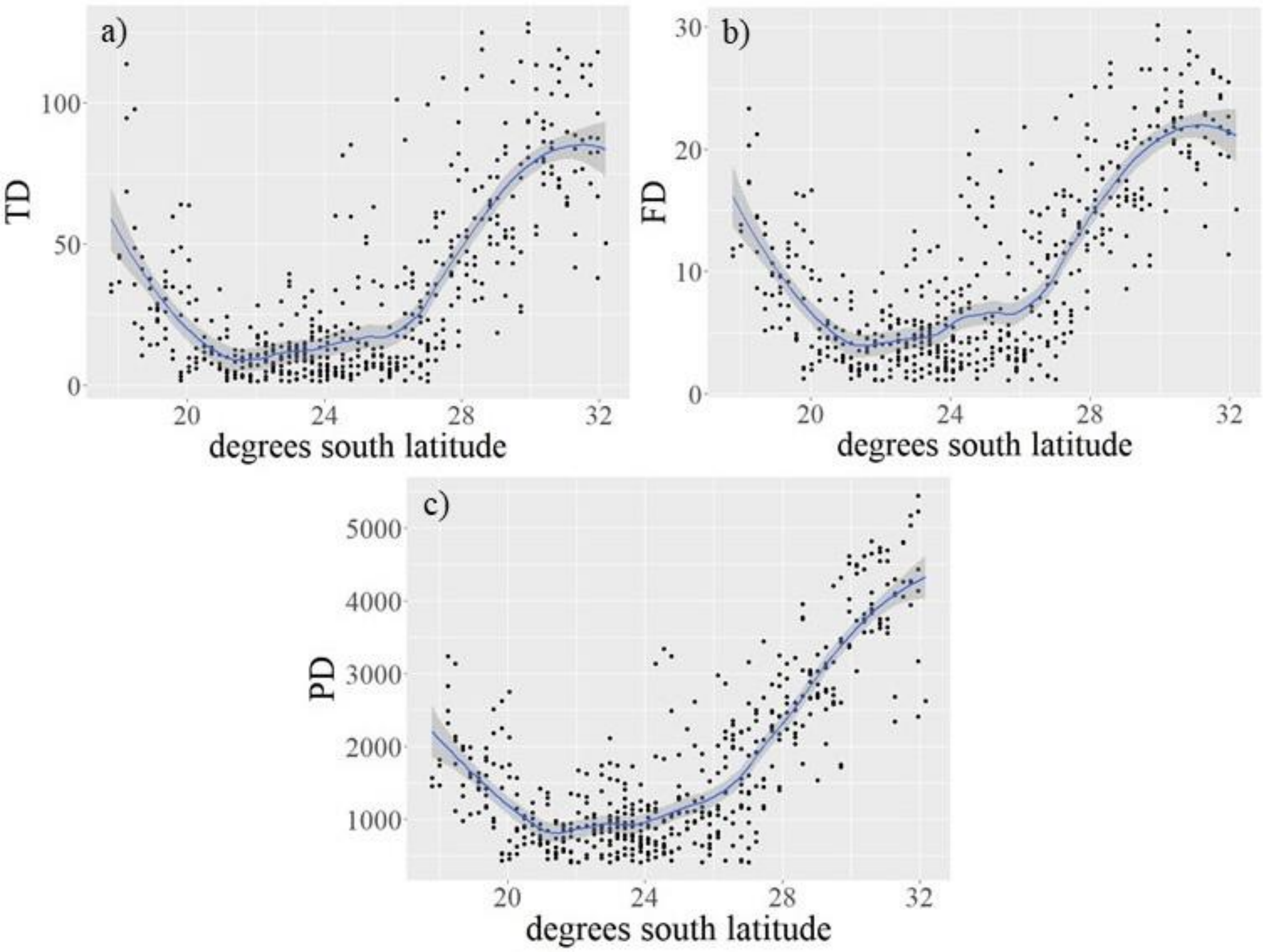

Fig. S6. Latitudinal gradient of perennial flora diversity in the aridity gradient of Chile (a) Taxonomic (TD); (b) Functional (FD); and (c) Phylogenetic (PD).

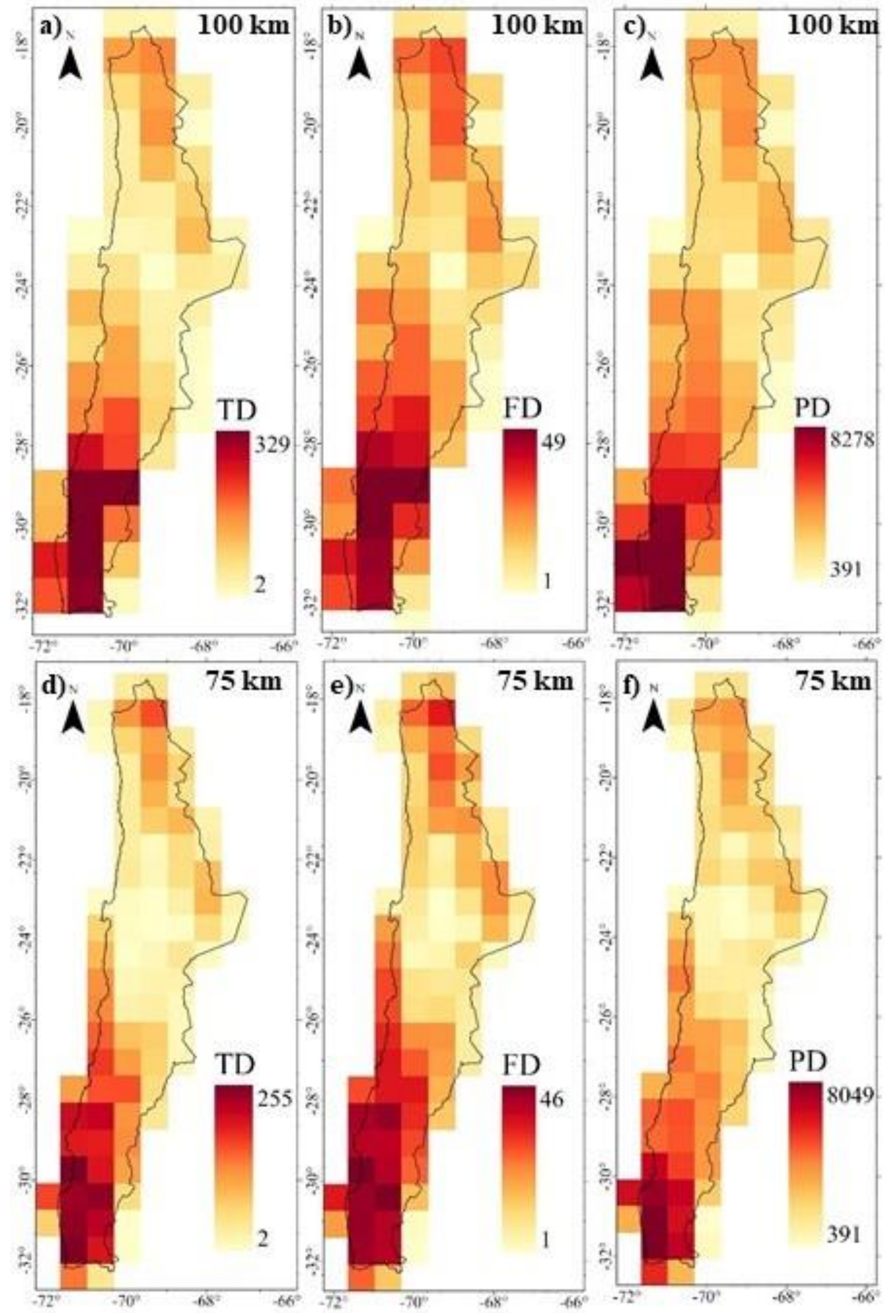

Fig. S7. Spatial patterns of diversity at different resolutions (100 and 75 km grid cells) of the perennial flora of northern Chile. a) TD with 100 km grid cell; b) FD with 100 km grid cell; c) PD with 100 km grid cell; d) TD with 75 km grid cell; e) FD with 75 km grid cell; and e) PD with 75 km grid cell.

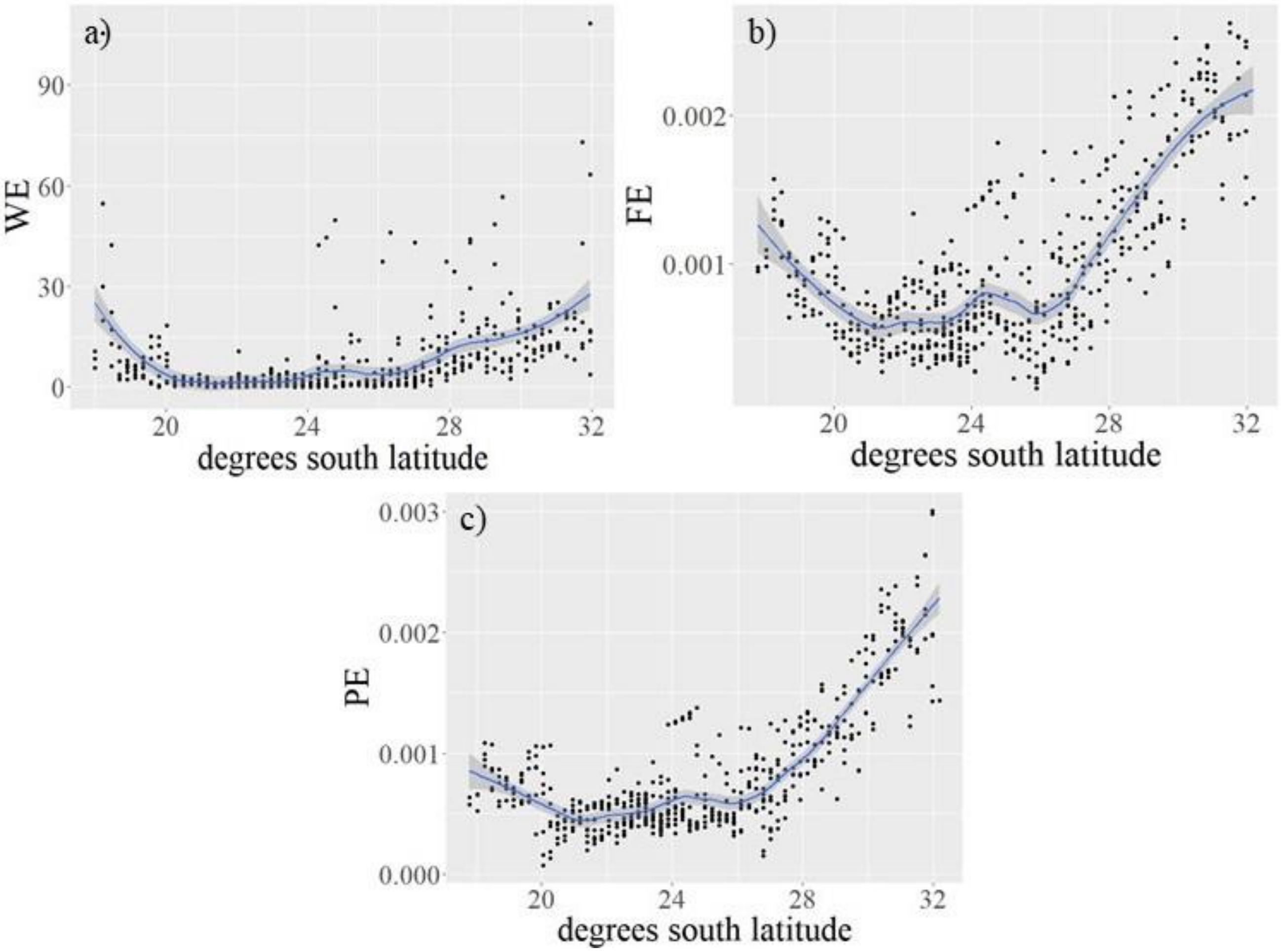

Fig. S8. Latitudinal gradient of the perennial flora endemism in the aridity gradient of Chile (a) Taxonomic (WE); (b) Functional (FE); and (c) Phylogenetic (PE).

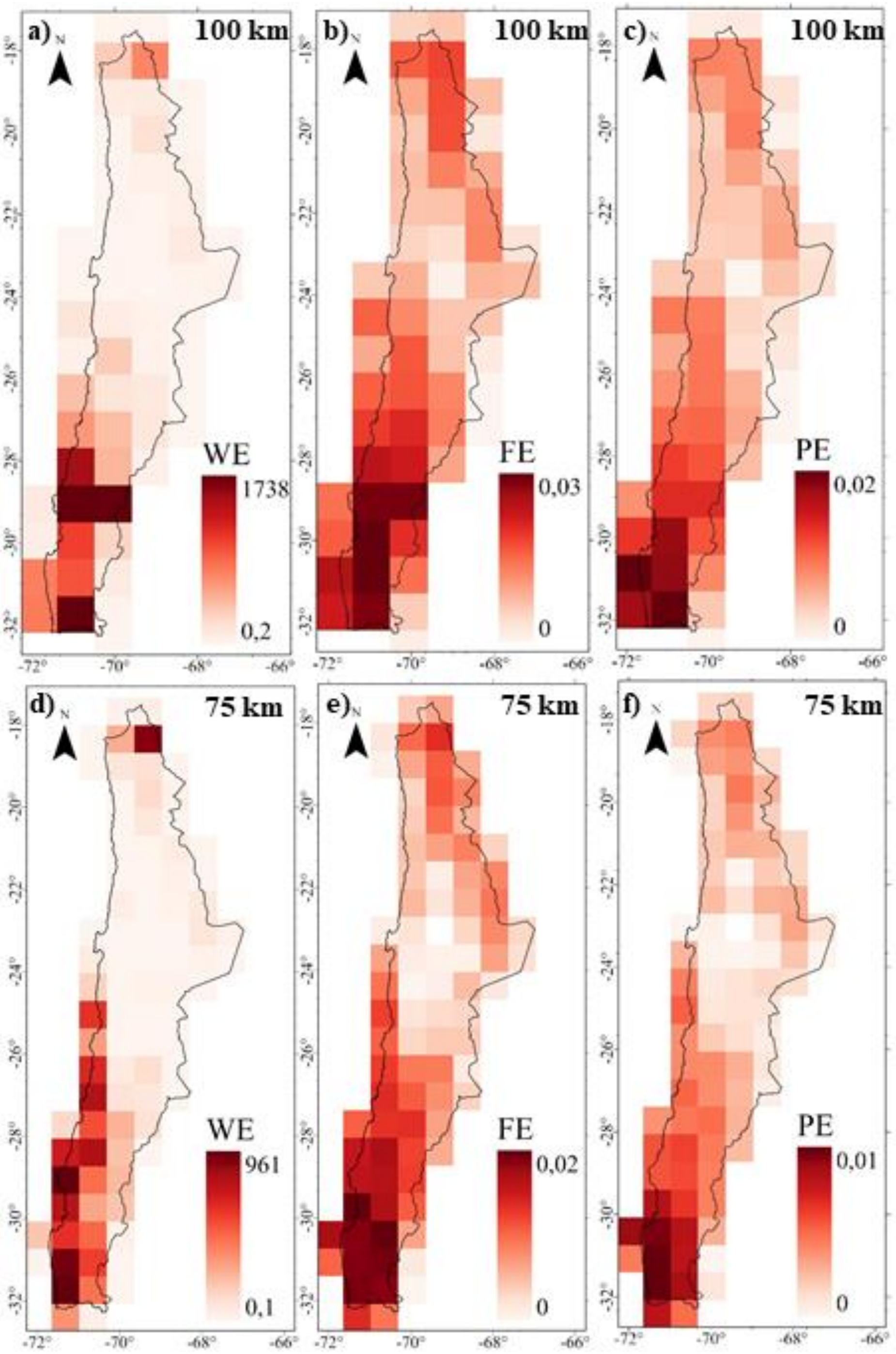

Fig. S9. Spatial patterns of endemism at different resolutions (100 and 75 km grid cells) of the perennial flora of northern Chile. a) WE with 100 km grid cell; b) FE with 100 km grid cell; c) PE with 100 km grid cell; d) WE with 75 km grid cell; e) FE with 75 km grid cell; and e) PE with 75 km grid cell.

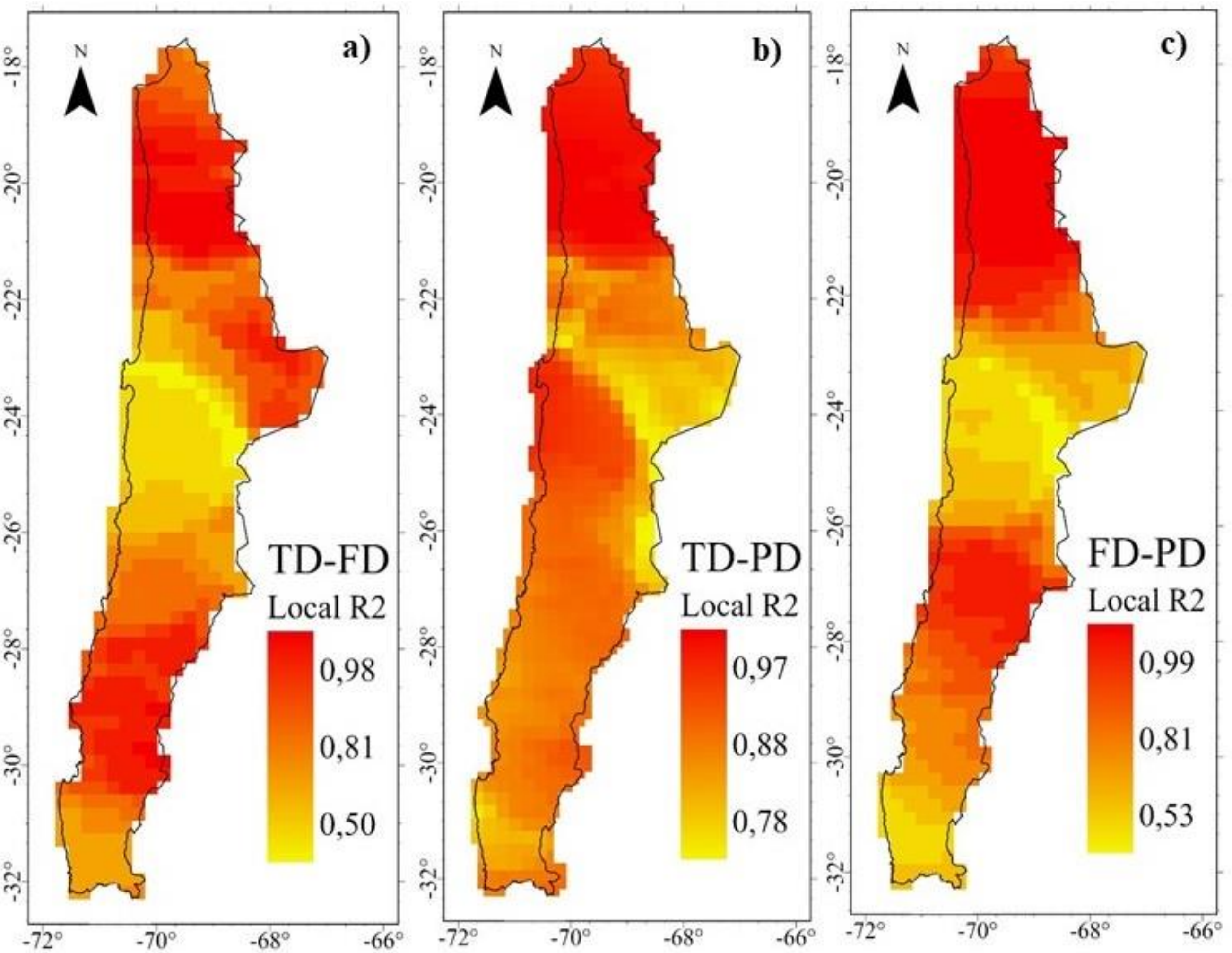

Fig. S10. Multiscale Geographically Weighted Regression (MGWR) of the diversity of the perennial flora of northern Chile. The colour schemes represent the local R^2 value: a) Taxonomic~Functional, b) Taxonomic~Phylogenetic, and c) Functional~Phylogenetic.

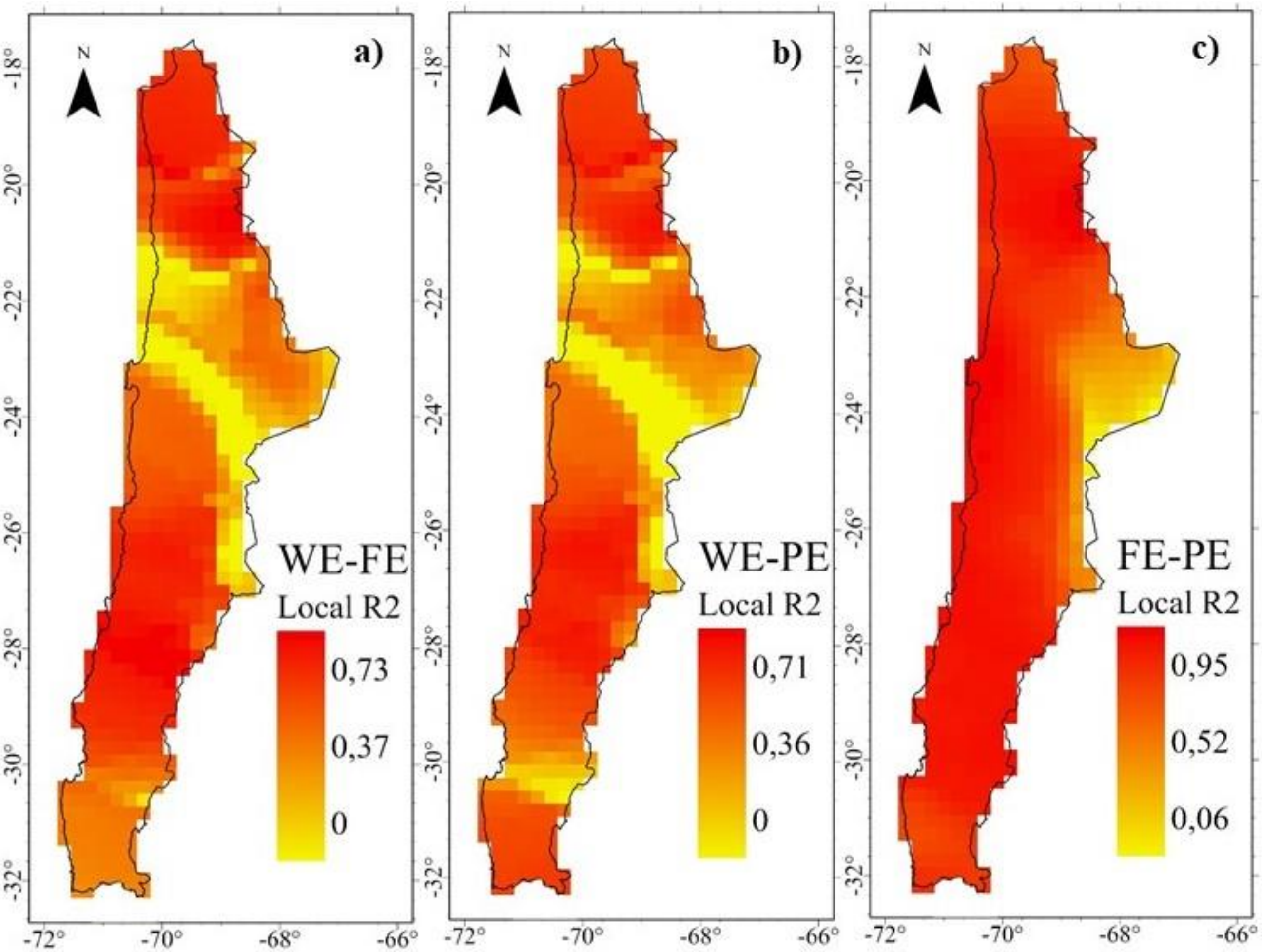

Fig. S11. Multiscale Geographically Weighted Regression (MGWR) of the endemism of the perennial flora of northern Chile. The colour schemes represent the local R^2 value: (a) Taxonomic ~ Functional, (b) Taxonomic~Phylogenetic, and (c) Functional~Phylogenetic.
